# First large-scale ethnobotanical survey in the province of Uíge, northern Angola

**DOI:** 10.1186/s13002-018-0238-3

**Published:** 2018-07-25

**Authors:** Thea Lautenschläger, Mawunu Monizi, Macuntima Pedro, José Lau Mandombe, Makaya Futuro Bránquima, Christin Heinze, Christoph Neinhuis

**Affiliations:** 1 Department of Biology, Institute of Botany, Faculty of Science, Technische Universität Dresden, 01062 Dresden, Germany; 2 University Kimpa Vita, Province of Uíge, Rua Henrique Freitas No. 1, Bairro Popular, Uíge, Angola

**Keywords:** Medicinal plants, Angola, Ethnobotany, Influence of distance, Gender-specific, Neophytes

## Abstract

**Background:**

Angola suffered a long-lasting military conflict. Therefore, traditional knowledge of plant usage is still an important part of cultural heritage, especially concerning the still very poor health care system in the country. Our study documents for the first time traditional knowledge of plant use of local Bakongo communities in the northern province of Uíge on a large scale with a focus on medicinal plants and puts data in context to different parameters of age, gender and distance to the provincial capital.

**Methods:**

Field work was carried out during nine field trips in 13 municipalities between October 2013 and October 2016. In 62 groups, 162 informants were interviewed. Herbarium specimens were taken for later identification. Database was analysed using Relative Frequency of Citations, Cultural Importance Index, and Informant Consensus Factor. Furthermore, significances of influence of age, gender and distance were calculated.

**Results:**

Our study presents 2390 use-reports, listing 358 species in 96 plant families, while just three out of 358 mentioned species are endemic to Angola about one-fifth are neophytes. The larger the distance, the higher the number of use citations of medical plants. Although women represent just a fifth of all citations (22%), their contribution to medicinal plants was proportionally even higher (83%) than those of men (74%). Fifty percent of all plants mentioned in the study were just listed by men, 12% just by women. We made some new discoveries, for example. *Gardenia ternifolia* seems to be promising for treatment of measles, and *Annona stenophylla* subsp. *cuneata* has never been ethnobotanically nor phytochemically investigated.

**Conclusions:**

While the study area is large, no significant influence of the distance in regard to species composition in traditional healer’s concepts of the respective village was pointed out. Although several plants were just mentioned by women or men, respectively, no significant restriction to gender-specific illnesses in medical plant use could be found. Merely concerning the age of informants, a slight shift could be detected.

**Graphical abstract:**

Visual representation of the ethnobotanical study in Uíge, northern Angola.
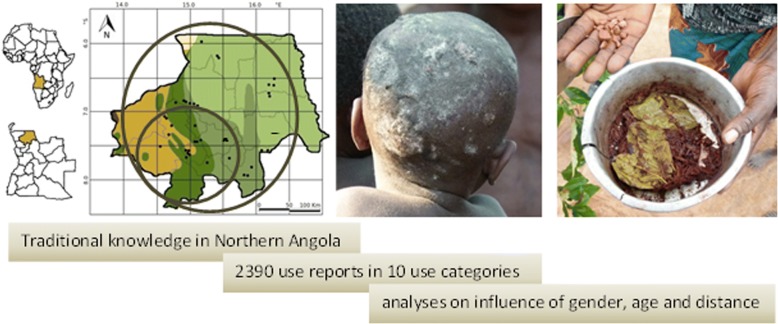

**Electronic supplementary material:**

The online version of this article (10.1186/s13002-018-0238-3) contains supplementary material, which is available to authorized users.

## Background

Angola is regarded as a country with an unusually rich biodiversity covering a high amount of vegetation zones and habitats [1, 2]. Although several botanists, among them Friedrich Welwitsch (1806–1872), Hugo Baum (1867–1950) and John Gossweiler (1873–1952), visited and studied this richness, the war lasting 40 years did not allow them to carry out continuous botanical or ethnobotanical investigations [1]. Bossard (1987, 1993) investigated Ovimbundu traditional medicine, listing plant names just in Ovimbundu language without identifying botanical species [3, 4]. Nowadays, the considerable work of Figueiredo and Smith [1] creating a plant checklist for the country with about 7000 species represents a useful database for following and future studies. While quite a number of surveys were conducted in Southern Angola, just a few are located in the northern part [5, 6]. Göhre et al. [7] collected ethnobotanical data in disturbed areas around the city of Uíge. Monizi et al. [8] described a high variety of wild plants used for securing human survival in Ambuila, one of the 16 municipalities in the province of Uíge [8]. Heinze et al. [9] conducted the first ethnobotanical studies in the neighbouring province Cuanza Norte. Specific descriptions of fibre uses were given by Senwitz et al. [10]. According to the distribution of the ethnic tribe Bakongo, covering northern Angola as well as the adjacent Bas-Congo area, ethnobotanical studies conducted in the Democratic Republic of Congo should reveal comparable results of ethnobotanical uses in Angola [11].

Traditional knowledge is essential for the healthy cultural and social life within a society [12]. It is generally assumed that indigenous traditional knowledge information is going to be lost because it is, at least partly no longer essential for the survival of people. This is either due to influences such as the rapid development of rural areas or because of displacement of indigenous people [13, 14]. Although several infrastructure measures were undertaken in Angola, development is still slow, especially regarding the public health sector. Even if child mortality in Africa decreased during the last two decades, it is still very high. More specifically, Angola has the highest rate in Africa and worldwide and, following Sierra Leone the lowest life expectancy for women and men worldwide [15, 16]. Sousa-Figueiredo et al. [17] detected malnutrition and anaemia as public health problems. Smith et al. [18] documented that the overall prevalence of malnutrition is higher in rural than then in urban areas. In this context, ethnobotanical studies in northern Angola seemed reasonable not only in terms of documentation of the current state but urgently needed to record still existing knowledge. Furthermore, Moyo et al. [19] stated that the rich flora of sub-Saharan Africa suggests enormous potential for discovery of new chemical components with therapeutic value.

In our large-scaled survey in the northern province of Uíge, covering about 60,000 km^2^, 13 out of 16 municipalities were visited, including both savannah and forest formations. Therefore, this study for the first time

1. Provides an overview of traditional plant uses and health methods in the province of Uíge
2. Highlights native as well as introduced plant species used in traditional medicine
3. Analyses the influence of gender, age and distance from the province capital Uíge with regard to uses and methods

## Methods

### Study area

**Fig. 1:**
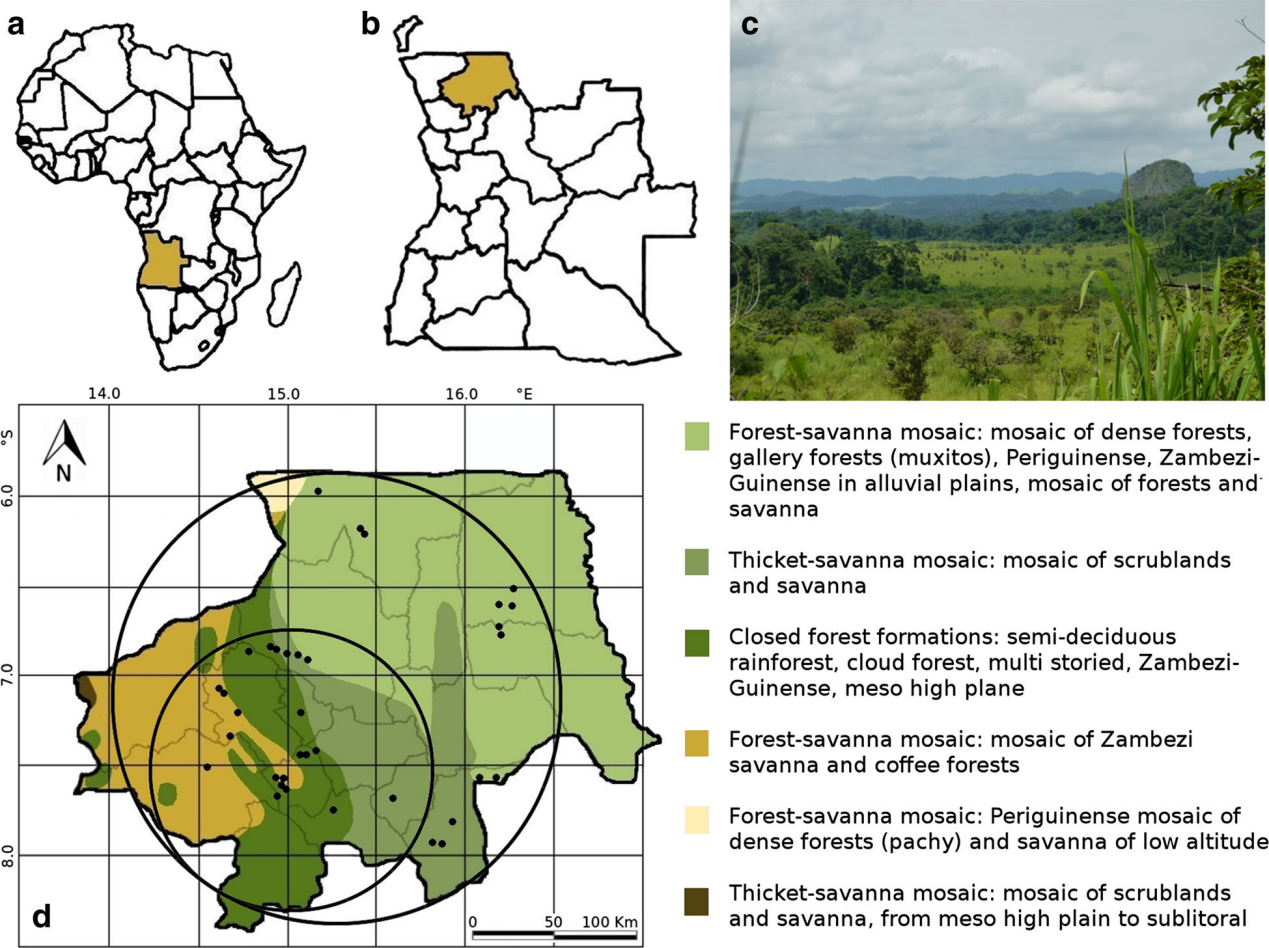
**a** Location of Angola in Africa, **b** province of Uíge in Angola, **c** mosaic of forest and savannah patches in the municipality of Ambuila (**d**) map of study area with vegetation zones, collection sites marked with a black dot and circles representing the distance to Uíge city: inner circle ≤ 160 km, outer circle > 160 km; vegetation zones according to Barbosa [26]. Carta fitogeográfica de Angola. Instituto de Investigação Científica de Angola, Luanda. Graphic: Andreas Kempe

The studies were conducted in the province of Uíge located in the very north of Angola, bordering in the north and east to the Democratic Republic of the Congo, in the south to the provinces of Malanje, Cuanza Norte, and Bengo, and in the west to Zaire province (Fig. 1). According to the Köppen climate classification, the province has a tropical wet or dry or savannah climate Aw [20, 21]. This so-called Guineo-Congolian rainforest climate is characterized by a rainy season lasting at least 6 months, relative air humidity above 80% and typical dense fog, locally called *Cacimbo* [22–24]. Due to the global ecoregions map defined by the World Wildlife Fund (WWF), the province of Uíge covers the ecoregion called the Western Congolian Forest-Savannah Mosaic [25]. A more precise description of the region was given by White [24] who classified Angola north between the Guineo-Congolian and the Zambesian Regions, i.e., the Guinea-Congolian/Zambesian Regional Transition Zone. According to that classification, this zone is characterized by a high complexity since elements of both formations are present. Edaphic conditions and the existence of a diverse topography strongly influence the formation of distinctive patterns of mosaic vegetation shown in Fig. 1c. Barbosa [26] subdivided the area into six vegetation zones, shown in Fig. 1d.

The long lasting war in Angola had a highly negative impact on biodiversity [27]. But also prior to the conflicts, several species of economic value on international timber markets like *Milicia excelsa* (Welw.) C.C.Berg or species of *Entandrophragma* were historically exploited and are still under increasing pressure [22]. This rising forest loss is confirmed by global analysis of satellite data [28]. Moyo et al. [19] calculate for Guinean Forests in West Africa a remaining area of 15%. On the other hand, the National Report on forest resources by the FAO [29], based on data captured by Horsten, reported not more than 4% of the Uíge area as productive [7, 30]. Beside deforestation, Göhre et al. [7] reported uncontrolled burning caused by growing agricultural activities. Hence, large areas are heavily disturbed anthropogenically resulting in an increased abundance of Zambezian floristic elements following the destruction of the original vegetation leaving only secondary grass- and woodland [24]. Recordings in the remaining forest patches exhibit tropical rainforest and savannah species assemblages comparable with the Bas-Congo region [11, 31, 32].

Since the vegetation formations are very heterogeneous, traditional use of plants by people is prevalent and manifold. The province comprises 16 municipalities, covering an area of 58.698 km^2^ inhabited by more than 1.4 million people [33], the majority of which belongs to the Kikongo speaking Bakongo ethnic group [33]. As this Bantu group is also living in the neighbouring countries Democratic Republic of the Congo, Republic of the Congo, and Gabon, manifold influences caused by migration due to political problems and conflicts are part of its culture. Very little is known about the health care system in Angola. Faith-based organizations’ contribution to Angola’s health care system is very low, compared to other sub-Saharan countries [34]. In turn, the government is cutting the health budget due to the falling prices for oil [35]. The lack of health infrastructure, especially in rural areas, is a serious problem resulting in the constant importance of traditional healers and herbal medicines [36].

### Data collection

Data sampling was carried out between 5° 58′ 59.2″ and 7° 56′ 59.4″ southern latitude and between 14° 33′ 53.7″ and 16° 17′ 04.5″ longitude, covering 35 localities in 13 municipalities (Fig. 1). According to the distance from the provincial capital Uíge, two distance levels A (≤ 160 km) and B (170–330 km) were defined. During nine field trips between October 2013 and October 2016, 162 informants were involved in the study, 30 of those were interviewed on their own, 132 were interviewed in groups of two to five persons, bringing the total number of interviews to 62. In advance, the University Kimpa Vita formulated credentials to inform the mayors of the municipalities about the planned activities. To establish contact with potential informants, local authorities of the visited villages (called *soba* and *seculo*) were informed about the aims and methods of the study and asked to suggest persons with experience in traditional medicine that might participate (prior informed consent). Hence, all the interviews were conducted with at least one traditional herbalist sometimes accompanied by laypeople. We tried to form a gender-balanced research without violating cultural and/or sacred taboos [37]. The specification of the obtained knowledge varied from location to location and person to person. Information was collected during semi-structured interviews, transect walks and group discussions [38]. Criteria used to define the uses reported are based on informant’s statements. Since Silva et al. [39] recommended vegetation inventories to guarantee a correct identification of species and better identification by informants, walks into the traditionally used plant collecting areas were always part of interviews, including forest and savannah formations, since these two habitats alternate very frequently. During field-work, Portuguese language was mainly used, however, in some cases, Angolan colleagues translated into Kikongo. Gender and age of every informant was documented wherever possible. In those cases where the informant did not know his exact age, it was estimated whether the person was younger or older than 40. The following data sets were requested: local plant name, its usage, used plant part and preparation techniques. In case of medicinal plants administration techniques were also documented. Local market surveys and field trips for collecting herbarium specimens completed the investigations. All processes of the surveys were permitted and accompanied by the local authorities. Following the advice made by Ramirez [14] to allow a better contribution and exchange of knowledge, we invited several informants to our presentations and discussions at the University Kimpa Vita in Uíge city. The code of ethics of the International Society of Ethnobiology was followed. The study was carried out in compliance with the agreement of Access and Benefit Sharing. For identification, plants were photographed and plant voucher specimens were collected, dried and stored at the Dresden herbarium (Herbarium Dresdense), Technische Universität Dresden, Germany. In a Memorandum of Understanding between the Instituto Nacional da Biodiversidade e Áreas de Conservação (INBAC), Angola and the Technische Universität Dresden, Germany, signed in 2014, it was agreed upon that duplicates will be returned to Angola as soon as appropriate conditions to store the herbarium vouchers are established. The Ministry of Environment Angola and the Province Government of Uíge issued the required collection and export permits. Identification of collected plant specimens and data analysis was completed in Dresden, Germany. For identification, several floristic works were used: Conspectus Florae Angolensis [40], Plantas de Angola [1], Flore Analytique du Bénin [41], Flora of Tropical West Africa [42–46], and Flora Zambesiaca [47]. Additional information was retrieved from Kew Herbarium Catalogue [48] and Naturalis Biodiversity Center [49]. Furthermore, for some plant families, specialists were consulted. The Herbario LISC and Herbario COI were visited in July 2016 and 2017 for comparing plant samples [50]. Use-reports of identified plants were only included in the results if the specimen was at least determined to genus level. The nomenclature used refers to Plantlist.org. Voucher specimen numbers of Herbarium Dresdense as well as photo voucher numbers are given in Table 1. Due to the poor availability of data regarding the information of endangered species, Table 1 includes only additional details on endemism and states of neophytes.

**Table 1:** Overview of all collected and identified useful plants from the Province Uíge: Species listed alphabetically; additional information on usage, used plant part (PP), preparation and administration, use category (UC), number of citations and number of informants. Species information provided: Origin: E = endemic; + = listed; (-) = not listed; * = naturalised according to Plants of Angola (Figueiredo and Smith, 2008); vernacular names in Portugues (Port.) and Kikongo (Kik.); Voucher number according to Herbarium Dresdense or Foto voucher (F); Plant parts: B = bark, BU = bulb, F =fruit, FL = flower, L = leaf, LA = latex, MY = mycel, R = root, RE = resin, RH = RH, S = seed, SS= stem sap, ST = stem, W = whole plant, WO = wood; Use Category: C = drugs and cigarettes, D = domestic and charcoal, F = hunting and fishing, H = handicrafts, L = ludic, M = medicine, N = nutrition, O = other, R = ritual, T = dental care and cosmetics

Species	Usage	PP	Preparation	Administration	UC	Citations	Informants
*Abelmoschus esculentus* (L.) Moench*,* Kiabo (Port.), Kingombo (Kik.), Kiabua*,* F_01	Diabetes	L	Decoction	Oral	M	1	2
	Diarrhea	L	Decoction	Oral	M	1	
	Intestinal inflammation	L	Decoction	Oral	M	1	
	Nutrition	F			N	1	
*Abrus precatorius* L.*,* Dinzenze, Dienguele (Kik.)*,* 44228	Activates lactation	L	Crudité	Oral	M	2	2
	Cough	L	Crudité	Oral	M	1	
	Erectile dysfunction	L	Crudité	Oral	M	1	
*Abutilon fruticosum* Guill. & Perr.*,* Lunzunzu lua mpembe (Kik.) Ndondondo, 43828	Costal pain	L	Balm	Dermal	M	1	2
	Support birth (faster)	L	Decoction	Oral	M	1	
*- Acanthospermum glabratum* (DC.) Wild, Matata, Madiata (Kik.), 43361	Infection legs	L	Balm	Dermal	M	1	3
	Migraine	W	Roast, Pulverize	Dermal	M	1	
	Open fontanelle (baby, old people)	L	Balm	Dermal	M	1	
	Skin disease	L	Balm	Dermal	M	1	
	Yellow fever	L	Balm	Dermal	M	1	
*Acanthospermum hispidum* DC., Madiatadiata (Kik.), 42727	Skin disease	L		Dermal	M	2	2
*Acanthus montanus* (Nees) T.Anderson, Kekasanga*,* Nkeka ngô (Kik.), Indulumba, sosongui, 43375	Angina pectoris	L	Decoction	Oral	M	1	7
	Epilepsy	R	Decoction		M	1	
	Hepatitis	L			M	1	
	High blood pressure	L	Decoction	Oral	M	1	
	Infertility	L	Roast, Pulverize	Oral	M	3	
	Leg pain	L	Crudité	Dermal	M	1	
	Nutrition	L			N	1	
	Scoliosis	L	Decoction	Bath, Dermal	M	2	
	Stomach pains	L		Enema	M	1	
*Adansonia digitata* L., Imbondeiro (Port.), Nkondo (Kik.), Mucua, F_02	Lemonade	F			N	1	1
	Skin disease	L	Balm	Dermal	M	1	
*Adenia cissampeloides* (Planch. ex Hook.) Harms, Nkawu (Kik.), 45030	Infertility women	R			M	1	1
*Adenia lobata* (Jacq.) Engl., Mukekete, Nkenkete (Kik.), Muloa, 43834	Epilepsy	SS	Crudité	Eyes Drops	M	1	5
	Nutrition	L		Oral	N	4	
*Aframomum alboviolaceum* (Ridl.) K.Schum., Gingengue (Port.), Mansasa, Mansansa ma londe, Manzunja, Ntundulu (Kik.), Linguenga, dizaza Xinguenga, Mazasa da queimada, mazaza gingenga, nzaza, Ntundabala, Kizaza, 44161	Antibiotic	R	Maceration	Oral	M	1	16
	Bloody urin	R	Maceration		M	1	
	Constipation	R	Decoction	Enema	M	1	
	Convulsion	L, SS	Crudité	Nose Drops	M	1	
	Diabetes	R	Decoction	Hip Bath	M	1	
	Epilepsy	R, ST	Decoction, Maceration, Crudité	Enema, Bath	M	4	
	Epilepsy	L	Percolation	Eye Drop	M	2	
	Hernia	R		Enema	M	1	
	Inflammation legs	R	Crudité	Bath	M	2	
	Low blood pressure	R	Maceration	Oral	M	1	
	Nutrition	F			N	6	
	Parsitic worms	R	Maceration	Oral	M	1	
	Scoliosis	L	Decoction	Enema, Bath, Dermal, Oral	M	8	
	Sterility (men and women)	R	Decoction	Oral	M	1	
	Stomach pains	R	Maceration	Oral	M	1	
	Vertiz	ST	Decoction	Face Wash	M	1	
	Yellow fever	R			M	1	
*Aframomum angustifolium* (Sonn.) K.Schum., Gingenga da mata (Port.), Mansasa ma mfinda (Kik.), F_04	Nutrition	F			N	2	2
	Yellow fever	R			M	1	
*Aframomum melegueta* K.Schum., Ndungu za kongo (Kik.)*,* 44226	Mixture component	S			M	1	3
	Spice	S			N	2	
* Agave sisalana Perrine, Fibra de sisal (Port.), Barabate (Kik.), F_05	Fiber plant	L			F	1	2
	Rope	L			H	1	
*Agelaea pentagyna* (Lam.) Baill., Kamatatu (Kik.), 42832		R			M	1	1
		L			M	1	
*Agelanthus brunneus* (Engl.) Tiegh., Nkunda nkunda (Kik.), 43338	Eye infection	L	Percolation	Eye drops	M	1	2
	Stomach pains	L	Decoction	Enema	M	1	
*Ageratum conyzoides* (L.) L., Fuatakala, Imbuakatela (Kik.), 43160	Vertigo				M	1	1
*Albizia adianthifolia* (Schum.) W.Wight, Mulu (Kik.), mulukai, 44243	Bleeding	R	Decoction	Enema	M	1	11
	Cold	L	Percolation	Nose Drops	M	1	
	Construction	WO			D	1	
	Cough	L	Crudité	Oral	M	1	
	Epilepsy	R	Decoction, Maceration	Enema	M	2	
	Eye parasites	R	Percolation	Eye Drops	M	5	
	Fodder plant	L			F	1	
	Headache	R	Percolation	Nose Drops	M	1	
	Hemorrhoids	L	Decoction	Enema	M	1	
	Infidelity of father	L	Roast, Pulverize	Oral	R	1	
	Malaria	L	Percolation	Nose Drops	M	1	
	Nosebleed	R	Percolation	Nose Drops	M	2	
*Albizia ferruginea* (Guill. & Perr.) Benth., Makaba, Nsuemba (Kik.)*,* 44220	Epilepsy	B	Decoction	Nose Drops	M	1	2
	Fodder plant	L			F	1	
*Alchornea cordifolia* (Schumach. & Thonn.) Müll.Arg., Bunza, Gunze, Wunze (Kik.), kibunge,mbunzi, Kimbunza, Guunze, Muunze, lumbunze, Kiunzia, 42586	Anaemia	L	Decoction	Oral	M	1	12
	Anaemia	B	Decoction	Oral	M	1	
	Bloody diarrhea	R, L	Decoction	Oral	M	2	
	Decoration graveyard	F			R	1	
	Diarrhea	L, B	Decoction	Oral	M	2	
	Eye pain	R	Percolation	Eye Drops	M	2	
	Fire wood	WO			D	1	
	Hemorrhoids	L	Suppository	Rectal	M	1	
	Hunting birds	F			F	1	
	Open cervix	L	Balm	Anal, Vaginal	M	2	
	Otitis	L	Percolation	Ear Drops	M	1	
	Parasites in eyes	R	Percolation	Eye Drops	M	1	
	Skin disease	L, B	Balm, Decoction	Dermal, oral	M	3	
	Surgery wounds	L	Infusion	Oral	M	1	
	Toothache	L, B	Decoction	Oral	M	4	
	Weakness	L, B	Decoction	Oral	M	2	
*- Allium sativum* L., Alho (Port.), F_06	Infertility	BU	Decoction	Oral	M	2	2
	Stomach pains	BU	Decoction	Oral	M	1	
*Allophylus africanus* P.Beauv., Mbanzu mbanzu (Kik.), 41878	Fodder plant	L			F	2	2
*Aloe buettneri* A.Berger, Ba dia Nseke (Kik.), kikalango, ndende, 43280	Cough	L	Crudité	Oral	M	1	6
	Erectile dysfunction	ST	Decoction	Oral	M	1	
	Gonorrhea	L, SS	Crudité	Vaginal	M	1	
	Headache	L	Balm	Dermal	M	1	
	Hernia	R	Decoction	Enema	M	1	
	Mixture component	L			M	1	
	Splenomegaly	L	Decoction	Enema	M	2	
*Alvesia rosmarinifolia* Welw., Mazima-zima, Mfinguila (Kik.), 43910	Open fontanelle (baby, old people)	L	Balm	Dermal	M	1	2
	Pain	L, ST	Infusion	Dermal	M	2	
	Vertigo	L	Balm	Dermal, Nose Drops	M	2	
* Amaranthus caudatus L., Biteku teku, Bowa (Kik.), Gimboa, 43908	Nutrition	L			N	2	2
* Anacardium occidentale L., Cajú, Cajueiro (Port.), Nkazuwa (Kik.), F_06	Nutrition	F, S			N	4	3
	Vertiz	B	Decoction	Bath	M	1	
* Ananas comosus (L.) Merr., Abacaxi (Port.),Nanazi (Kik.), F_07	Backache	F	Fermentation	Oral	M	1	1
*Anchomanes difformis* (Blume) Engl., Nsadia kiula (Kik), 44160	Earache	ST	Put into Fire	Emitted Spume into Ear	M	1	2
	Splenomegaly	BU	Decoction	Inhalation	M	1	
*Aneilema beninense,* Mpimpita (Kik.), 42713	Nutrition	L			N	1	1
*Anisophyllea quangensis* Engl. ex Henriq., Mfungua (Kik.), mfuongo, Ifungu, xifungu, 43266	Cough	L	Decoction	Oral	M	1	7
	Eye parasites	R	Percolation	Eye Drops	M	1	
	Lung problems	L	Decoction	Oral	M	1	
	Nutrition	F			N	6	
	Nutrition	L			N	1	
	Scoliosis	R	Decoction	Dermal	M	2	
*- Anisophyllea sororia* Pierre*,* lufuongo*,* F_09	Nutrition	F			N	1	1
*- Annona muricata* L., SSi SSi (Port.), Mbundu a ngombe (Kik.), 44055	Nutrition	F			N	1	1
*Annona senegalensis* Pers., Lolo kIambulu*,* Lolo (Kik.)*,* F_10	Bloody diarrhea	R	Decoction	Enema	M	1	3
	Nutrition	F			N	1	
	Stomach pains	L, R	Decoction, Infusion	Oral	M	4	
*Annona stenophylla* subsp. *cuneata* (Oliv.) N.Robson, Lolo*,* Lolo kia ndamba, Nlolo a mpolo*,* Nzelenge (Kik.), nolopolo, muloloa, Loloalolo, mulolo, molo, Nlolo kafioti, lolonbulu, malolo, dilolo, 43204	After loss of pregnancy	R	Maceration	Enema	M	1	23
	Anaemia	L	Decoction, Infusion	Oral, Bath	M	5	
	Anaemia	R	Decoction	Oral	M	1	
	Appendix	R	Maceration	Enema	M	1	
	Backache	L, R	Decoction	Oral	M	2	
	Cleaning stomach	L, R	Decoction	Oral	M	2	
	Constipation	R	Decoction, Maceration	Enema	M	2	
	Cryptorchidism	R	Maceration	Oral, Enema	M	2	
	Diarrhea	R	Decoction	Enema	M	1	
	Epilepsy	R	Decoction, Maceration	Enema	M	2	
	Hemorrhoids	R	Decoction	Oral	M	1	
	Hernia	R	Maceration	Oral, Enema	M	2	
	Infertility women	R	Decoction		M	1	
	Influenza	R	Maceration	Bath	M	1	
	Malaria	R	Maceration	Bath	M	1	
	Nutrition	F			N	13	
	Open cervix	L			M	1	
	Parasitic worms	R	Maceration	Oral	M	1	
	Scoliosis	R, L	Decoction	Dermal	M	2	
	Stomach pains	R	Decoction, Maceration	Oral	M	4	
	Tea	L	Infusion		N	2	
	Typhus	L	Decoction, Infusion	Oral	M	2	
*- Antidesma laciniatum* var. *Membranaceum* Müll.Arg., Munzevo nzevo (Kik.) 43259	Bleedings	F	Decoction	Oral	M	1	1
*Antidesma venosum* E.Mey. ex Tul., Mfutila (Kik.), 43868	Skin disease	F	Swallowing		M	1	1
*- Artocarpus altilis* (Parkinson ex F.A.Zorn) Fosberg, Fruta pão (Port.), Santu Petelo (Kik.), 42674	Nutrition	F			N	1	1
*Asparagus drepanophyllus* Welw. ex Baker, Nlandu, Timba timba (Kik.), malekatanga, F_11	Cryptorchidism	BU	Chewing		M	1	2
	Strong menstruation	BU			M	1	
*Asparagus laricinus* Burch., Mandioca (Port.), Dioko dia nkama, Nsensa mpakasa, Nzezangoma (Kik.), 44003	Backache	R			M	1	4
	Cough	BU	Crudité	Oral	M	1	
	Erectile dysfunction	BU	Decoction	Enema, Dermal	M	2	
	Headache	BU	Balm	Dermal	M	1	
	Infertility (male)	BU	Eat	Oral	M	1	
	Menstruation (severe)	RH	Decoction	Enema	M	1	
	Nosebleed				M	1	
	Stomach pains	BU	Decoction	Enema	M	1	
*Asparagus* spec., Nsesa mpakasa (Kik.)*,* 44737	Cough	BU	Chewing, Cook	Oral	M	2	3
	Erectile dysfunction	BU	Crudité, Maceration In Palm Wine	Oral	M	3	
*- Azadirachta indica* A.Juss., Neem, 44233	Stomach pains	L			M	1	1
*Baccharoides guineensis* (Benth.) H.Rob., Matita, Nkokomakioko, Nsakaba (Kik.), 43279	Baso	BU	Crudité	Enema	M	1	9
	Body pain	BU	Balm	Dermal	M	1	
	Burns	L	Balm	Dermal	M	1	
	Constipation	BU	Pulverize	Enema	M	1	
	Cough	BU	Crudité	Oral	M	1	
	Diarrhea	BU	Maceration	Oral	M	1	
	Headache	BU	Balm	Dermal	M	1	
	Inflammation testicles	BU	Decoction	Enema, Dermal	M	2	
	Injury	BU	Balm	Dermal	M	1	
	Lack of appetite	BU	Crudité	Oral	M	1	
	Sprain	BU	Balm, Chewing, Tie Around Belly	Dermal, Oral	M	5	
	Stomach pains	BU	Crudité, Decoction	Oral, Enema	M	4	
	Trap for mole	BU			F	1	
*- Bambusa vulgaris* Schrad., Bamboo (Port.), Tutu dia mputu (Kik.), F_13	Construction	ST			D	2	3
	Erosion control	W			O	1	
*- Baphia chrysophylla* Taub. Mbidimbidi, Mbidi (Kik.) Ntandambínza F_14	Bloody urin	R	Decoction	Enema	M	1	2
	Urinal infection	R	Maceration	Oral	M	1	
*Barteria nigritana* Nzumizumi (Kik.) 42749	Skin disease	L	Decoction	Dermal	M	1	1
*Basella alba* L. F_16	Nutrition	L			N	1	1
*Bauhinia thonningii* Schum. Pata do boi (Port.), Nsakala (Kik.) loloa, musakala 43847	Diabetes	R, SS		Oral	M	1	8
	Diarrhea	B	Decoction	Oral	M	1	
	Hemorrhoids	B	Decoction	Oral	M	1	
	Open cervix	B	Crudité, Decocton	Vaginal, Oral, Bath	M	3	
	Tool handle	WO			D	2	
	Typhus	B	Decoction	Oral	M	1	
	Weakness after birth	B	Decoction	Bath	M	1	
*Bidens pilosa* L. Kimananganzi, Kolokoso (Kik.) 42743	Tea	L			N	1	2
		W			M	1	
*Biophytum umbraculum* Welw. Zambakonono (Kik.) 43236	Chills	W	Decoction	Enema	M	1	2
	Weakness	W	Decoction	Bath	M	1	
*Bobgunnia madagascariensis* (Desv.) J.H.Kirkbr. & Wiersema Nzuku (Kik.) muzuku, nsambozeke 43829	Fever	B	Maceration	Enema	M	1	2
	Open fontanelle	L	Balm	Dermal	M	1	
	Rattle	F			L	1	
* Boerhavia diffusa L. Ditumbato (Kik.)	Hepatitis	W	Maceration	Enema	M	1	3
	Malaria	W	Maceration	Enema	M	1	
	Malaria	L			M	1	
	Snakebite	L			M	1	
	Yellow fever	L	Decoction	Bath	M	1	
*Brachystegia spiciformis* Benth. Kwidi, Nkuidi (Kik.) 44141	Nutrition	WO			N	1	1
	Tobacco	B			C	1	
*Brassica* spec. Couve (Port.), Nkove (Kik.) 42794	Nutrition	L			N	1	1
*Brenandendron donianum* (DC.) H.Rob. Mundala ndala (Kik.) 44115	Headache	L	Balm, Maceration	Dermal	M	2	4
	High fever children	L	Decoction	Bath	M	1	
	Package	L			O	1	
*Bridelia ferruginea* Benth. Mukalakala*,* Mwindu, Nkangati (Kik.), munkangati, mukala 44197	Anaemia	B	Decoction	Oral, Enema	M	2	15
	Bleeding	R	Decoction	Enema	M		
	Bloody diarrhea	B, R	Decoction	Oral	M	3	
	Construction	WO			D	1	
	Cough	B			M	1	
	Diarrhea	L	Crudité	Oral	M	1	
	Diarrhea	B	Maceration	Oral	M	1	
	Dysentery	R	Decoction	Oral	M	1	
	Fodder plant	L			F	2	
	Headache	B	Smoking		M	1	
	Healing wounds	B	Incinerate	Dermal	M	1	
	Injury	B	Balm	Dermal	M	3	
	Injury	R	Balm	Dermal	M	1	
	Open cervix	B	Crudité	Vaginal	M	1	
	Stomach pains	R	Decoction	Oral	M	1	
	Strong menstruation	B	Maceration	Oral	M	1	
	Tobacco	B	Incinerate	Nose Drops	C	2	
	Weakness	R	Crudité	Oral	M	1	
*Bridelia micrantha* (Hochst.) Baill. Minzundu, Mukalakala da mata (Kik.) 44224	Fodder plant	L			F	6	6
*Brillantaisia owariensis* P.Beauv. Lemba lemba, Malembalemba (Kik.) 44259	Against storms (totosimalembosi)	L			R	1	11
	Burn injuries	L	Incinerate	Dermal	M	1	
	Crying baby	L, R	Put into Cradle	Dermal	M	2	
	Epilepsy	L	Maceration	Dermal	M	1	
	Growth from the stump	L			D	1	
	Headache	L	Infusion, Maceration	Oral, Dermal	M	4	
	Heart problems	L	Infusion	Oral	M	2	
	High blood pressure	L			M	1	
	Infertility women	L	Roast	Oral	M	1	
	Madness	R, L	Percolation	Nose Drops	M	2	
	Many uses	L	Infusion		M	1	
	Solves problems	L			R	1	
	Stomach pains	L	Infusion	Oral	M	2	
	Stress	L			M	1	
	Struck by lightning	L	Crudité	Oral	M	2	
	Tachycardia	L			M	1	
*- Brugmansia versicolor* Lagerh. F_17	Insect bite	L	Balm	Dermal	M	1	1
	Snake bite	L	Balm	Dermal	M	1	
*Burkea africana* Hook. Kilobo (Kik.) 44200	Fodder plant	L			F	3	3
* Cajanus cajan (L.) Millsp., Mbwengwe*,* Wando (Kik.), Muando, feijao uandu 44033	Nutrition	S			N	3	4
	Parasites in eyes	L	Percolation	Eye Drops	M	1	
*Calamus deerratus* G.Mann & H.Wendl., Junco (Port.), Mbamba (Kik.) 44125	Construction	ST			D	1	2
	Rattan	ST			H	2	
*Calvoa seretii* De Wild., Nzikinseke (Kik.) 43333	Nutrition	L	Decoction		N	1	1
*Canarium schweinfurtii* Engl., Kimfwabidi, Mbidi, Mfuambidi (Kik.), Obafu, mumbidi, mbafu F_18	Asthma	RE	Decoction	Oral	M	1	8
	Backache	B		Enema	M	1	
	Candle	RE			O	2	
	Cough	RE	Burn Incense	Inhalation	M	2	
	Energy source	RE			D	1	
	Fodder plant	L			F	1	
	Incense	RE			R	1	
	Mixture component	RE			M	1	
	Nutrition	F			N	3	
	Parasitic worms	RE	Sucking	Oral	M	1	
	Stomach pains	RE	Pulverize, Sucking	Oral	M	2	
		B			M	1	
*- Canavalia gladiata* (Jacq.) DC., Feijão (Port.), Madezo, Nkasa (Kik.) F_19	Nutrition	S	Cook	Oral	M	1	1
* Canna indica L. 42701	Pain when breathing	R	Crudité	Dermal	M	1	2
	Toys	S			L	1	
- *Capsicum annuum,* Ndungu za kongo, Ndungu za matebo (Kik.), 42694	Hemorrhoids	F	Decoction	Enema	M	1	3
	Madness	F	Percolation	Nose Drops	M	1	
	Nutrition	F			N	1	
* Capsicum frutescens, Gindungu (Port.), Ndungu (Kik.) F_21	Nutrition	F			N	3	3
* Carica papaya L., Mamão (Port.), Papayi (Kik.) F_22	Backache	L	Crudité	Anal	M	1	14
	Backache	R	Decoction	Oral	M	1	
	Bloody urin	R	Maceration		M	1	
	Caries	R	Decoction	Mouth Wash	M	1	
	Diabetes	R	Decoction	Oral	M	1	
	Diarrhea	R			M	2	
	Diarrhea caused by breast milk	L	Maceration	Oral	M	1	
	Fertility men	R	Decoction	Oral	M	1	
	Flatulence	L	Balm	Dermal	M	1	
	Gonorrhoea	R	Decoction	Oral	M	1	
	Hemorrhoids	R	Balm, Decoction	Dermal, Enema	M	2	
	Induced abortion	R	Decoction	Oral	M	1	
	Injury	R	Balm	Dermal	M	1	
	Madness	R	Percolation	Nose Drops	M	1	
	Potency	R	Decoction	Oral	M	1	
	Toothache	R	Decoction	Mouth Wash	M	3	
	Typhus	R	Decoction	Oral	M	1	
	Yellow fever	R	Decoction	Oral	M	1	
*- Catharanthus roseus* (L.) G.Don, 43937	Amoeba	R	Decotion	Oral	M	1	3
	Beauty, decoration	FL			R	1	
	Decoration	FL			R	1	
*Ceiba pentandra* (L.) Gaertn., Mfuma (Kik.), mufumeira, Kapok 42781	Anaemia	B	Decoction	Oral	M	1	2
	Canoe construction	WO			H	1	
* Celosia argentea L., sankokolo 43935	Decoration	FL			R	1	1
	Nutrition	L			N	1	
*Celosia trigyna* L. 43173	Epilepsy	L, R	Maceration	Enema	M	2	2
	Nutrition	L			N	1	
*Celtis gomphophylla* Baker, Munzunzua mfinda (Kik.) 44219	Fodder plant	L			F	1	1
*Cereus* spec., Nsoma (Kik.) F_23	Lightning conductor	W			O	1	1
*Ceropegia bonafouxii* K.Schum. 43253	Skin disease	L	Balm		M	1	1
*Chaetocarpus africanus* Pax, Kosu kosu, Nkovola, Nkungui a nteka*,* Nkunguteka (Kik.) 43229	Animal trap	ST			F	1	5
	Cough	L	Infusion	Oral	M	1	
	Fungi on skin	F, L	Balm	Dermal	M	2	
	Lepra	L	Decoction	Enema, Dermal	M	2	
	Mpungu = bad magic (inflammation arms, genital area)	L	Decoction		M	1	
*Chassalia cristata* (Hiern) Bremek., nlotunlotu F_74	Skin disease	R	Balm	Dermal	M	1	1
	Skin parasites	R	Balm	Dermal	M	1	
*Chlorophora excelsa* (Welw.) Benth.*,* Moreira (Port.), Nkamba (Kik.), murere 44231	Construction	WO			D	3	4
	Infection	ST			M	1	
*- Chromolaena odorata* (L.) R.M.King & H.Rob.*,* Kongo ya sika, Mubutu (Port.)*,* mabutu, wasabanga, kabukila, Nguengele, Ntumisina 42706	Backache	L	Crudité	Dermal	M	1	17
	Body pain	L	Pulverize	Dermal	M	1	
	Cold	L	Decoction	Inhalation	M	1	
	Fever	L	Balm	Dermal	M	1	
	Flu	L	Infusion	Oral	M	1	
	High fever	L	Decoction	Oral, Steam Bath	M	2	
	Injury	L	Balm	Dermal	M	6	
	Injury	L	Pulverize	Oral	M	1	
	Malaria	L	Decoction	Oral, Inhalation, Enema	M	5	
	Soil improvement	W			O	1	
	Stomach pains	L	Decoction	Oral	M	2	
	Stomach pains (reason: dirty water)	L	Maceration	Oral	M	1	
	Wounds	L	Balm, Tincture	Dermal	M	3	
*Chrysophyllum* cf*. bangweolense* R.E.Fr.*,* Ngonti, Nkosi nti, Ntele (Kik.), muhonga 44156	Constipation	R	Decoction	Enema	M	2	4
	Hernia	R	Decoction	Enema	M	1	
	Leg ache	R	Balm	Dermal	M	1	
	Parasitic worms	R	Decoction	Enema	M	1	
	Snakebite	R	Balm	Dermal	M	1	
	Splenomegaly children	R	Balm	Dermal	M	1	
	Stomach pains	R	Decoction	Enema	M	2	
*Cissus rubiginosa* (Welw. ex Baker) Planch, Faz tudo (Port.), Katambadi, Nkatakata kahendanga, Nkata mbadi, Nkokila mbundu (Kik.), holamo zunzu 42591	Arm pain	ST	Crudité	Dermal	M	1	12
	Bloody diarrhea	L	Decoction	Enema	M	1	
	Hernia	R		Enema	M	1	
	Inflammation	L	Balm	Dermal	M	1	
	Inflammation testicles	BU	Decoction	Enema, Testicles	M	2	
	Injury				M		
	Leg pain	ST	Balm, Crudité	Dermal	M	2	
	Nutrition	F			N	1	
	Red eyes	L	Decoction, Percolation	Oral, Eye Drops	M	2	
	Rheumatism	F	Balm	Dermal	M	1	
	Skin disease	R	Balm	Dermal	M	1	
	Sprain	F	Balm	Dermal	M	1	
	Stomach pains	R	Decoction	Oral	M	1	
	Struck by lightning	L	Balm	Dermal	M	1	
	Struck by lightning	R		Enema	M	1	
	Yellow fever	L	Decoction	Oral	M	1	
*- Citrus reticulata* Blanco*,* Tangerineira (Port.)	Lemonade	F		Oral	N	1	1
	Tea	L	Infusion	Oral	N	1	
*Citrus* spec., Limão (Port.), Lala dia nsa (Kik.)	Bloody urin	R	Maceration		M	1	4
	Cake	F			N	1	
	Diabetes	R	Decoction	Oral	M	1	
	Gonnorrhe	R	Decoction	Oral	M	1	
	Lemonade	F			N	1	
*- Citrus* x *limon* (L.) Osbeck, Limão (Port.)	Cold	F		Dermal	M	1	4
	Cough	F	Crudité, Decoction	Oral	M	2	
	Desinfection	F		Dermal	M	1	
	Fertility men	F	Crudité	Oral	M	1	
	Fertility men	R	Decoction	Oral	M	1	
	Skin disease	FL	Balm	Dermal	M	1	
*- Citrus* x *sinensis* (L.) Osbeck, Laranjeira (Port.) F_28	Angina	L	Decoction	Oral	M	1	1
	Fever	L	Decoction	Oral	M	1	
*- Clematis hirsuta* Guill. & Perr.*,* Feijao maluco (Port.), Mankundia (Kik.), Mutsiatsia 43350	Headache	L	Percolation	Nose Drops	M	1	1
*- Clematis uhehensis* Engl. 44009	Puppet	F			L	1	1
*Clematis villosa* DC., Zalandemba (Kik.) 44167	Epilepsy	R	Maceration	Oral	M	1	1
*Clerodendrum formicarum* Gürke, Kinda ngolo, Nlombua mvula (Kik.), Nlombamvula 43286	Infection legs	L	Balm	Dermal	M	1	4
	Leg ache	L	Decoction	Dermal	M	1	
	Skin disease	L	Balm	Dermal	M	1	
	Stomach pains	L	Decoction	Enema	M	1	
	Urinal infection	L, R		Oral	M	2	
*Clerodendrum fuscum* Gürke, Maculu (Port.), 44213	Anus infection	L	Incinerate	Dermal	M	1	1
	Mouth infection	L	Incinerate	Dermal	M	1	
*Clerodendrum splendens* G.Don, Kindangolo (Kik.)	Parasitic worms	L	Decoction	Oral	M	1	1
	Stomachache	L	Decoction	Oral	M	1	
	Weakness	L	Decoction, Maceration	Oral	M	2	
*Clerodendrum welwitschii* Gürke, Ndia a nzamba (Kik.) 43254	Otitis	R			M	1	1
*Clitandra cymulosa* Benth.*,* Madinga, Makalanga (Kik.) 43183	Nutrition	F			N	2	2
	Parasitic worms	LA	Crudité	Enema, Oral	M	2	
*Cnestis corniculata* Lam., Kizikizamba (Kik.) 43886	Allergic shock	R	Maceration	Oral	M	1	1
*Cnestis ferruginea* Vahl ex DC. 43890	Nutrition	F			N	1	1
*- Coffea robusta* L.Linden Café (Port.), Kafi (Kik.) F_30	Toothache	R	Decoction	Mouth Wash	M	1	1
*Cogniauxia podolaena* Baill.*,* Mpakambai, Mazakanbulu 42751	Chills	L	Decoction	Oral	M	1	3
	Menstrual cramps	R		Enema	M	1	
	Splenomegaly	R	Decoction	Enema	M	1	
*Cola acuminata* (P.Beauv.) Schott & Endl.*,* Cola (Port.), Makazu (Kik.), Nkazu brasa 41880	Aphrodisiac agent	S	Pulverize	Oral	M	1	7
	Back pain	B	Decoction		M	1	
	Backache	S	Pulverize	Oral	M	1	
	Diabetes	L	Decoction	Oral	M	1	
	Diarrhea	S	Crudité	Eat	M	1	
	Hemorrhoids	S	Crudité	Oral	M	1	
	Open fontanelle	S	Balm	Dermal	M	1	
	Ritual	S			R	1	
	Stimulants	S			C	1	
	Weakness	S	Pulverize	Oral	M	1	
*Colletoecema dewevrei* (De Wild.) E.M.A.Petit*,* Nzekazeka (Kik.) F_31	Cough	L	Chewing	Oral	M	1	1
	Injury	B	Balm	Dermal	M	1	
*- Colocasia esculenta* (L.) Schott*,* Batata malanga (Port.), Malanga (Kik.) F_32	Nutrition	RH			N	2	2
*Combretum celastroides* Welw. ex M.A.Lawson*,* Nzenze (Kik.) 43978	Sore throat	R	Decoction	Inhalation	M	1	1
*Combretum collinum* Fresen.*,* mugiti 43881	Construction	WO			D	1	2
	Hemorrhoids	L	Balm, Decoction	Dermal, Enema	M	2	
*Combretum psidioides* Welw., Nkukuti (Kik.) 44215	Backache	R	Apply On Surface	Dermal	M	1	6
	Bloody diarrhea	L	Decoction	Oral	M	1	
	Bronchitis	F	Pulverize	Oral	M	1	
	Construction	WO			D	1	
	Diarrhea	L, ST	Decoction	Enema	M	2	
	Hemorrhoids	B	Balm, Roast	Anal	M	2	
	Hemorrhoids	R	Crudité	Anal	M		
	Skin disease	B	Balm, Decoction	Dermal, Bath	M	3	
	Stomach pains	L	Balm	Oral	M	1	
*Combretum racemosum* P.Beauv.*,* Nsumbila, N'sumbi, Nsumbi a mbi (Kik.), nsumbiele 44794	Bloody diarrhea	L	Decoction, Maceration	Oral	M	2	6
	Bloody diarrhea	R	Maceration	Oral	M	1	
	Diarrhea	L	Maceration	Oral	M	1	
	Dysentery	L	Decoction	Oral	M	1	
	Stomach pains	R	Decoction	Enema	M	1	
*Commelina diffusa* Burm.f., Kambuakatela (Kik.) 44029		R			M	1	1
*Costus afer* Ker Gawl.*,* Makenia*,* Mankene, Matumba tumba*,* Nsangalavu*,* Nsangalavua*,* (Kik.) 46847	Activates lactation	ST	Chewing	Oral	M	1	14
	Cleaning uterus and testicles	ST			M	1	
	Cough	L	Chewing	Oral	M	1	
	Enuresis	ST	Chewing		M	1	
	Epilepsy	ST	Chewing	Eye Drops	M	4	
	Epilepsy	L	Maceration	Dermal	M	1	
	Eye problems	ST	Percolation	Drop In Eye	M	2	
	Gout	W	Maceration	Enema	M	1	
	Insomnia	W	Maceration	Enema	M	1	
	Mixture component	L	Chewing	Oral	M	1	
	Nutrition	L, ST			N	2	
	Parasites in eyes	ST	Percolation	Eye Drops	M	1	
	Trypanosomiasis	F		Oral	M	1	
	Weakness	ST	Chewing		M	2	
	Yellow fever	ST	Chewing	Eye Drops	M	1	
*Costus spectabilis* (Fenzl) K.Schum.*,* Longa di nseke, Longa dia simbi*,* Ntesi ntesi (Kik.) 42652	Erectile dysfunction	RH	Chewing, Maceration	Oral, Enema	M	3	4
	Eye problems	RH	Percolation	Eye Drop	M	1	
	Water belly	L			M	1	
*Crassocephalum montuosum* (S.Moore) Milne-Redh., Bungudi (Kik.)*,* Bungula 43282	Nutrition	L			M	1	1
*Crassocephalum rubens* (Juss. ex Jacq.) S.Moore*,* Bungudi (Kik.)*,* bungudia 44082	Chest pain	L	Cook	Eat	M	1	5
	Nutrition	L			N	4	
*Crinum* spec., Bá dia nseke (Kik.) F_34	Yellow fever	BU	Decoction	Enema	M	1	1
*Crossopteryx febrifuga* (Afzel. ex G.Don) Benth.*,* Mvala (Kik.)*,* nhala, Vala 43907	Baso children	R		Enema	M	1	10
	Cleaning body	R	Decoction	Enema	M	1	
	Cold	R			M	1	
	Constipation	R	Crudité	Enema	M	1	
	Diabetes	R		Enema	M	1	
	Epilepsy	R	Decoction	Nose Drops	M	1	
	Fertility men	R	Decoction	Oral	M	1	
	Flatulence	R	Crudité, Decoction	Enema	M	2	
	Gonorrhoea	B	Decoction	Enema	M	1	
	Harelip	R	Percolation	Nose Drops	M	1	
	Headache	L	Decoction	Washing	M	1	
	Headache	R	Percolation	Nose Drops	M	1	
	Madness	R, L	Percolation	Nose Drops	M	3	
	Malaria	R	Percolation	Nose Drops	M	1	
	Scoliosis	L	Decoction	Dermal	M	1	
	Sexual potency	R	Decotion	Enema, Oral	M	2	
	Splenomegaly	L		Enema	M	1	
*Croton mubango* Müll.Arg.*,* Mbangu mbangu (Kik.) 44230	Backache	B		Enema	M	1	10
	Bodypain	B	Decoction	Oral, Dermal	M	2	
	Bone pain	B, L	Maceration	Enema, Bath	M	4	
	Epilepsy	L	Percolation	Nose Drops	M	1	
	Flu	B	Decoction	Steam Bath	M	1	
	Fodder plant	L			F	2	
	Joint pain	B, L	Maceration	Enema, Bath	M	4	
	Malaria	B			M	1	
	Scoliosis	B	Balm	Enema, Bath	M	2	
	Scoliosis	L	Balm	Enema, Bath	M	2	
	Shadowtree for coffee plantations	W			O	1	
	Stomach pains	B	Decoction, Maceration	Enema, Oral	M	2	
	Toys	S			L	1	
	Weakness	B		Enema, Dermal	M	2	
*Croton sylvaticus* Hochst., Ndianga, Nsonia (Kik.) 43127	Urinal infection	B	Crudité	Oral, Enema, Anal	M	3	1
*Cryptolepis oblongifolia* (Meisn.) Schltr.*,* Mukonki (Kik.) 44131	Detoxifying	L, R	Chewing, Put Behind Ear		M	3	2
	Erectile dysfunction	R	Chewing	Oral	M	1	
*- Cucurbita maxima* Duchesne*,* Muengeleka, Muteta (Port.), Lenge (Kik.), Kosekelenge F_35	Headache	F	Balm	Dermal	M	1	5
	Nutrition	L, F, S			N	4	
	Vertigo	F	Incinerate	Nose Drops	M	1	
*Culcasia angolensis* Welw. ex Schott, Mazanzala ngongolo (Kik.) 44133	Package	L			O	1	2
	Snake bite	L	Percolation	Dermal	M	1	
* Cymbopogon citratus (DC.) Stapf*,* Sinde (Kik.) 42882	Appetizing	L	Decoction	Oral	M	1	2
	Aromatization	L	Decoction	Oral	N	1	
*Cymbopogon densiflorus* (Steud.) Stapf*,* Lunsansangu (Kik.)*,* luzango 43869	Anorexia	F	Decoction	Oral	M	1	2
	Spice	FL	Decoction	Oral	N	1	
	Stomach pains	FL	Incinerate	Oral	M	1	
*Cyperus articulatus* L., Nianga za nkoko, Nsaku nsaku, Tangawisi (Kik.), Capi do Tangauisi, Usakusaku, Nlunianganu nloki 43939	Backache	RH		Enema	M	1	6
	Erectile dysfunction	RH			M	1	
	Foot infection	RH	Decoction	Enema	M	1	
	Stomach pains	RH		Enema	M	1	
	Toothache	RH	Tooth Balm		M	1	
		RH	Maceration	Oral	M	1	
		RH			M	1	
*Cyperus papyrus* L., Papiros (Port.), Mabu (Kik.) F_36	Mat	ST			H	4	4
*Cyperus* spec.	Rope	ST			H	1	1
^*E*^ *CyphoSTma stipulaceum* (Baker) Desc.*,* Nlembuzu (Kik.) 44139	Sore throat	R	Balm	Dermal	M	1	1
*Dacryodes edulis* (G.Don) H.J.Lam*,* N´safu (Kik.) F_37	Anaemia	L	Decoction	Bath, Oral	M	2	3
	Nutrition	F			N	1	
	Toothache	R, L	Decoction	Mouth Wash	M	2	
^*E*^ *Dalbergia carringtoniana* Sousa*,* hela 44174	Epilepsy	R	Percolation	Eye Drops	M	1	1
	Epilepsy	L	Percolation	Eye Drops	M	1	
	Stomach pains	R, L	Percolation	Eye Drops	M	2	
*Dalbergia nitidula* Baker, Katete (Kik.) 44135	Scoliosis	R	Decoction	Dermal	M	1	1
	Sprain	R	Decoction	Dermal	M	1	
*Daniellia alsteeniana* P.A.Duvign., Mulomba (Kik.) 44210	Construction	WO			D	1	3
	Erectile dysfunction	R	Chewing	Oral	M	1	
	Scoliosis	R	Decoction	Bath	M	1	
	Stomach pains	ST	Decoction	Oral	M	1	
	Toothache	B	Decoction	Mouth Wash	M	2	
	Weakness	R	Decoction	Bath	M	1	
*Daniellia klainei* A.Chev.*,* Nlomba (Kik.) 43982	Eye infection	FL	Crudité	Eye Drops	M	1	1
	Eye pain	FL	Crudité	Eye Drops	M	1	
* Datura metel L., tebu, Trampuapuasó 43938	Drug	S	Crudité	Oral	C	1	2
	Drug	L	On Hand	Dermal	C	1	
	Hallucinogenic	S			C	1	
* Datura stramonium L. F_38	Rheumatism	L	Pulverize	Dermal	M	1	1
*Desmodium setigerum* (E.Mey.) Harv.*,* Lunzila nzila, Luvuma*,* Mantata*,* Mungilagila (Kik.) 43826	Hemorrhoids	W	Decoction	Oral	M	1	4
	Open fontanelle	L	Balm	Dermal	M	1	
	Skin disease	L	Balm	Dermal	M	1	
	Splenomegaly	W	Balm	Dermal	M	1	
* Desmodium cf. *tortuosum* (Sw.) DC., Malama lama (Kik.) F_39	Antiaborting	L	Maceration	Enema	M	1	1
*Desmodium velutinum* (Willd.) DC. 43831	Infertility women	L	Cook	Eat	M	1	1
*Dialium englerianum* Henriq., Mbota, Mbota, Nsamba nzeke (Kik.)*,* Umbota 43176	Charcoal	WO			D	2	12
	Diarrhea	L	Chewing	Oral	M	1	
	Fodder plant	L			F	3	
	Headache	B	Decoction	Oral	M	1	
	Hiccup	B	Decoction	Oral	M	1	
	Lepra	B	Decoction	Enema, Dermal	M	2	
	Nutrition	F			N	6	
	Open fontanelle	B	Balm	Dermal	M	1	
	Parasitic worms	B, R	Maceration	Enema	M	2	
	Scoliosis	B	Balm	Dermal	M	2	
	Scoliosis	L	Decoction, Infusion	Dermal, Oral, Enema	M	5	
	Skin disease	B	Balm, Crudité	Dermal	M	2	
	Thrombosis	L, R	Decoction	Dermal	M	2	
	Toothache	B	Decoction	Inhalation	M	1	
*Dichrostachys cinerea* (L.) Wight & Arn.*,* Mvanga (Kik.) 44232	Anaemia	B	Balm	Dermal	M	1	2
	Fodder plant	L			F	1	
*Dioscorea BUifera* L.*,* Nsoko (Kik.) F_40	Nutrition	BU	Roast Slices		N	1	1
*Dioscorea praehensilis* Benth., Gindunga da mata, Inhame (Port.), Nsende za nkaka, Nsende za sadi, Sadi (Kik.), batata kisadi, mitoto 42602	Bloody diarrhea	BU	Cooked	Oral	M	1	8
	Constipation	ST	Decoction	Enema	M	1	
	Erectile dysfunction	R	Decoction	Oral	M	1	
	Infertility women	BU	Boiling	Enema	M	1	
	Nutrition	BU			N	3	
	Nutrition	ST			N	2	
	Sterility (men and women)	R	Decoction	Oral	M	1	
	Stomach pains	BU	Cooked	Oral	M	1	
	Stomach pains	R	Decoction	Oral	M	1	
*Diospyros heterotricha* (Welw. ex Hiern) F.White*,* Lufua lua ndombe, Munkonki (Kik.)*,* lufua 43975	Cosmetic	R			O	3	6
	Dental care	R	Chewing	Oral	T	4	
	Nutrition	F			N	1	
	Skin disease	F	Swallowing	Oral	M	1	
	Splenomegaly	R	Maceration	Enema	M	1	
	Toothache	R	Chewing	Oral	M	1	
*Diplorhynchus condylocarpon* (Müll.Arg.) Pichon*,* Mvondo ngolo, Vondisila, Nzo (Kik.)*,* Ihondegila, muhondixila, Insenge, Kisengi 42725	Construction	WO			D	1	11
	Diarrhea	LA	Crudité	Oral	M	1	
	Diarrhea	R	Decoction, Pulverize	Oral	M	2	
	Diarrhea children	R	Chewing, Decoction	Oral, Enema	M	2	
	Epilepsy	L	Decoction	Steam Bath	M	1	
	Gastrointestinal disease	R	Pulverize	Oral	M	1	
	Glue	LA			O	2	
	Mosquito repellent	LA	Crudité	Oral	M	1	
	Parasitic worms	R	Crudité, Maceration	Oral	M	2	
	Snake bite	LA	Crudité	Oral	M	1	
	Stomach pains	R	Chewing, Crudité, Decoction, Maceration	Oral, Enema, Dermal	M	5	
	Vertigo	L	Decoction	Dermal, Oral	M	3	
*Dissotis* spec., Mpangi mpangi*,* Nzangani (Kik.) F_41	Baso	L	Decoction	Enema, Oral	M	2	3
	Open fontanelle	L	Balm	Dermal	M	1	
*Dombeya burgessiae* Gerrard ex Harv. 43954	Rope	ST			H	1	1
*Dorstenia psilurus* Welw., Mikombo (Kik.) F_42	Back pain	R			M	2	6
	Chest pain	R	Decoction	Oral	M	1	
	Cough	R	Decoction	Oral	M	1	
	Infertility (male)				M	1	
	Malaria	R			M	1	
	Scoliosis	R	Decoction	Oral	M	1	
	So hide marihuana	R			C	1	
	Splenomegaly	S	Decoction	Enema	M	1	
	Stomach pains	R			M	2	
	Weakness	R	Maceration	Oral	M	1	
		R			M	1	
*Dracaena camerooniana* Baker, Nsalabakala (Kik.) F_43	Nutrition	L			N	2	2
*Dracaena mannii* Baker, Munzadi nzadi, Nsadisadi (Kik.), kitondo 44797	Angina	ST	Percolation	Oral	M	1	7
	Baby is crying a lot	ST	Percolation	Oral	M	1	
	Bird trapping	F			F	1	
	Fire WO	WO			D	1	
	Flu	L	Maceration	Bath	M	1	
	Headache	L	Balm, Maceration, Crudité	Dermal, Bath	M	3	
	Headache	B	Balm	Dermal	M	1	
	Headache	ST	Decoction		M	1	
	Open fontanelle	L	Balm	Dermal	M	1	
	Otitis	ST	Percolation	Ear Drops	M	1	
* Dysphania ambrosioides (L.) Mosyakin & Clemants, Santa Maria (Port.), Kinsidi nzimba (Kik.), kibuekete 42698	Backache	W	Crudité	Anal	M	1	14
	Bloody urin	R	Maceration		M	1	
	Cold	L	Decoction	Oral	M	1	
	Fever	W	Balm, Infusion	Dermal, Oral, Inhalation	M	4	
	Fever	L	Balm	Dermal	M	1	
	Flu	W	Decoction, Infusion	Oral, Bath, Inhalation	M	4	
	Flu	L	Decoction	Steam Bath	M	1	
	Headache	W	Decoction	Oral	M	1	
	Headache	L	Maceration	Dermal	M	1	
	Hepatitis	L	Crudité	Enema	M	1	
	Kidney problems	L	Maceration	Enema	M	1	
	Malaria	L	Balm	Dermal	M	1	
	Malaria	W	Decoction	Oral	M	1	
	Open cervix	L	Crudité	Enema	M	1	
	Open fontanelle	W	Balm	Dermal	M	1	
	Scoliosis	L	Decoction	Dermal	M	1	
	Vertigo	L	Maceration	Dermal	M	1	
	Yellow fever	L	Decoction	Bath	M	1	
*Ekebergia benguelensis* Welw. ex C.DC.*,* Fukamena nkosi (Kik.)*,* koxinti, Nfuka menakoxi, koxanti 43900	Constipation	R	Decoction	Enema	M	1	5
	Fever	R	Maceration	Enema	M	1	
	Parasitic worms	R	Maceration	Oral	M	2	
	Rheumatism	R	Balm	Dermal	M	1	
	Stomach pains	L	Decoction	Enema	M	2	
	Stomach pains	R	Decoction	Enema	M	2	
*Elaeis guineensis* Jacq., Dendê, Palmeira de dendê (Port.), Ngazi, Nkula, Nsamba, Nsoko a bá (Kik.), maruvu da palmeira, palmeira de dend, Kokonote F_44	Activates lactation	S	Chewing, Crudité	Eat, Oral	M	2	17
	Bloody diarrhea/dysentery	F, B	Pulverize	Oral	M	2	
	Bucket for peanuts	L			H	1	
	Cold (sniffles)	S	Oil	Nose Drops	M	1	
	Constipation	L	Decoction	Oral	M	1	
	Cryptorchidism	R	Decoction	Oral	M	1	
	Diarrhea	L	Crudité	Oral	M	1	
	Dysentery	B, F	Pulverize	Oral	M	2	
	Erectile dysfunction	R	Decoction	Oral	M	1	
	Eye parasites	F	Percolation	Eye Drops	M	1	
	Fish trap	L			F	2	
	Fodder plant	ST			F	1	
	Kidney problems	F	Maceration	Enema	M	1	
	Mixture component	F, S	Oil	Dermal	M	2	
	Nutrition	F, S			N	2	
	Palm wine	SS			C	3	
	Palm wine (prevents foaming)	S			O	1	
	Rip pain	F	Tie Around Body	Dermal	M	1	
	Ritual	FL, L			R	2	
	Splenomegaly	F	Decoction	Enema	M	1	
	Sprain	F	Balm	Dermal	M	1	
	Sterility (men and women)	R	Decoction	Oral	M	1	
	Stomach pains	S	Chewing	Oral	M	1	
	Stomach pains	R	Decoction	Oral	M	1	
	Stomachache	F	Crudité	Eat	M	1	
	Weak child	S	Oil	Dermal	M	1	
*Emilia coccinea* (Sims) G.Don, Lalulalu*,* Malalulalu (Kik.), Lanu 43903	Skin anomaly: Vitiligo	L	Maceration	Enema	M	1	3
	Skin disease	L	Balm	Dermal	M	2	
*Entada abyssinica* A.Rich.*,* Nsofi (Kik.) 43942	Asthma	L	Crudité	Oral	M	1	7
	Constipation	R	Decoction	Enema	M	1	
	Epilepsy	R	Decoction	Enema	M	1	
	Fodder plant	L			F	2	
	Headache	L	Balm	Dermal	M	2	
	Malaria	R	Percolation	Nose Drops	M	1	
	Parasitic worms	R	Decoction	Enema	M	1	
	Soap	R			O	1	
	Stomachache	R	Decoction	Enema	M	1	
*Eremospatha cuspidata* (G.Mann & H.Wendl.) H.Wendl., Junco (Port.), Lubamba, Mbamba (Kik.) 44128	Construction	ST			D	1	2
	Fish trap	ST			H	1	
*Eremospatha haullevilleana* De Wild.*,* Junco (Port.), Lubamba, Mbamba (Kik.) 44129	Constipation	ST	Decoction	Enema	M	1	4
	Dental care	ST			T	1	
	Fish trap	ST			F	1	
	Handicraft	ST			H	1	
	Penis infection	R	Decoction	Enema	M	1	
	Rattan	ST			H	1	
*Eremospatha hookeri* (G.Mann & H.Wendl.) H.Wendl., Mbamba (Kik.)*,* mabamba 44122	Construction	ST			D	1	1
*Eremospatha* spec., Junco (Port.) F_45	Animal trap	ST			F	1	1
*Eriosema glomeratum* (Guill. & Perr.) Hook.f., Bulukutu, Wandu wandu (Kik.), moando 43168	Fodder plant	L			F	1	3
	Tea	L			N	2	
	Vertiz	L	Maceration	Bath	M		
*Eriosema* spec.*,* Wando wando (Kik.)	Diarrhea	L	Crudité	Oral	M	2	3
	Fodder plant	L			F	1	
*Erythrina abyssinica* DC.*,* Lungu nlungu, Mungoma ngoma*,* Ngoma ngoma (Kik.), mulundulundu, mulungulungu 43909	Anaemia children	L	Decoction	Oral, Bath	M	2	15
	Back pain	B	Infusion, Decoction, Maceration	Oral, Dermal, Enema	M	4	
	Bloody diarrhea	R	Decoction	Enema	M	1	
	Coal	WO			D	1	
	Fodder plant	L			F	3	
	Hepatitis	B	Decoction, Maceration	Enema, Dermal	M	5	
	Irregular menstruation	R			M	1	
	Kidney	B			M	1	
	Open cervix	B		Hip Bath	M	1	
	Weakness	B	Crudité, Maceration	Oral	M	2	
	Yellow fever	B	Decoction, Maceration	Enema, Oral, Bath, Dermal	M	6	
	Yellow fever	R	Decoction		M	1	
*Erythrophleum africanum* (Benth.) Harms, Ngungu (Kik.), lugungu 43233	Caries	B, L	Decoction	Mouth Wash	M	2	7
	Charcoal	WO			D	1	
	Construction	WO			D	2	
	Displace rain	L			R	1	
	Fodder plant	L			F	6	
	Graveyard	WO			R	1	
	Leg pain	B	Balm	Dermal	M	1	
	Leg pain	R	Balm	Dermal	M	1	
	Menstruation problems	R	Decoction	Enema	M	1	
	Nosebleed	R	Decoction	Enema	M	1	
	Scoliosis	R	Maceration	Oral	M	1	
* Euphorbia hirta L., Kimvumina kia nkombo (Kik.) 43934	Amoeba in belly	W	Eat, Infusion	Oral	M	2	1
*Euphorbia tirucalli* L.*,* Muteta (Port.), Mbika (Kik.), Mbiku F_46	Supporting birth	LA		Dermal	M	1	1
*- Euphorbia tithymaloides* L. 42692	Decoration	FLs			R	1	1
*Fadogia cienkowskii* Schweinf., Mankindangolo (Kik.) 43830	Body pain	L	Balm	Dermal	M	1	2
	Stomach pains				M	1	
	Weakness	L	Balm	Dermal	M	1	
*Ficus bubu* Warb., Catato (Port.), Milenda (Kik.), milendemukua 44223	Fodder plant	L			F	1	1
*Ficus exasperata* Vahl, Nkuakasa (Kik.) 44166	Cleaning of dishes	L			O		1
*Ficus* spec., Nkuzu*,* Nsanga nsanga, Nsuemba (Kik.) F_76	Anaemia	L	Decoction	Oral	M	1	3
	Nutrition	F			N	1	
	Pilao (cassava pot)	WO			D	1	
	Weakness children	L	Balm, Decoction	Dermal, Oral	M	2	
*Ficus thonningii* Blume*,* N'sanda (Kik.), muzandi 44154	Rheumatism	B, L	Decoction	Bath	M	2	5
	Ritual	W			R	2	
	Support birth	B	Decoction	Oral	M	1	
	Symbol	W			R	2	
*Fleroya stipulosa* (DC.) Y.F.Deng*,* Nlongua (Kik.)*,* mulongo 43882	Constipation	R	Decoction	Enema	M	1	1
	Construction	WO			D	1	
*Gaertnera paniculata* Benth.*,* Nzuni nzuni (Kik.) 44173	Constipation	R	Decoction	Enema	M	1	2
	Scoliosis	L	Decoction	Dermal, Oral, Enema	M	3	
	Stomach pains	R	Decoction	Oral	M	1	
*Garcinia huillensis* Welw.*,* Kabobo (Kik.) 43854	Diarrhea	FL			M	1	1
	Nutrition	F			N	1	
*Garcinia kola* Heckel*,* Ngadiadia (Kik.) 44246	Infection legs	L	Balm	Dermal	M	1	6
	Intestine pain	S	Eat	Oral	M	1	
	Malaria	S	Chewing, Eat	Oral	M	4	
	Skin disease	L	Balm	Dermal	M	1	
	Snake bite	S	Chewing		M	1	
	Snake repellent	S			R	2	
	Stomach pains	S	Eat	Oral	M	1	
	Typhus	S	Chewing	Oral	M	2	
	Yellow fever	S	Chewing, Crudité	Oral, Enema	M	2	
*Gardenia ternifolia* subsp. *jovis-tonantis* Schumach. & Thonn., Nkia, Nkindia, Nlemba nzau (Kik.), ndai, Ndía 43245	Bloody diarrhea	B	Eat	Oral	M	1	13
	Debaso	F	Pulverize, Balm	Dermal	M	1	
	Diarrhea	B	Eat	Oral	M	1	
	Epilepsy	R	Percolation	Nose Drops	M	1	
	Joint pains	L	Decoction	Oral	M	1	
	Lightning conductor	L			R	1	
	Lightning conductor	F			O	1	
	Malaria	F	Pulverize, Balm	Dermal	M	1	
	Measles	F	Balm	Dermal	M	1	
	Measles	F	Crudité	Oral	M		
	Measles	S	Swallow	Oral	M	5	
	Pain	S			M	1	
	Parasites in mouth	F	Decoction	Mouth Wash	M	1	
	Prevent measles	S	Crudité	Eat	M	1	
	Stomach pains	L	Chewing	Oral	M	1	
	Stomach pains	B	Decoction	Oral	M	1	
	Toothache	F	Crudité	Oral	M	1	
	Typhus	S			M	1	
	Weakness	L	Infusion	Oral	M	1	
		S	Swallowing	Oral	M	1	
*Geastrum* spec. F_47	Urinal infection	MY	Decoction	Oral	M	1	1
	Weakness	MY	Decoction	Oral	M	1	
*Gladiolus dalenii* Van Geel*,* Malavu manuni (Kik.), kazeka nkuadi 44010	Backache	B		Enema	M	1	3
	Bird trap	FL			F	1	
	Urinal infection	BU	Maceration	Anal, Vaginak	M	2	
*Gloriosa superba* L.*,* Pisa maluca (Port.)*,* Dioko dia kiana, Mvia lawu (Kik.) 42734	Decoration	FL			R	1	6
	Male potency	RH			M	3	
	Parasitic worms	RH	Balm, Eat	Dermal, Orak	M	2	
	Stomach pains	RH	Balm, Eat	Dermal, Oral	M	2	
	Ximbasu = bad magic	RH	Balm, Eat	Dermal, Oral	M	2	
*Gnetum africanum* Welw., Mfumbwa (Kik.) 41876	Diabetes	L	Cooked	Eat	M	1	1
	Nutrition	L			N	1	
*Gongronema latifolium* Benth.	Antivenin	L	Chewing	Oral	M	1	1
	Bloody diarrhea	L	Chewing	Oral	M	1	
	Stomach pains	L	Chewing	Oral	M	1	
*- Gossypium barbadense* L., Algodão (Port.), Vusu (Kik.) 42693	Caries	R	Maceration	Mouth Wash	M	1	3
	Costal pain				M	1	
	Heartache	L	Decoction	Oral	M	1	
	Otitis	S	Oil	Ear Drop	M	1	
*Gymnanthemum glaberrimum* (Welw. ex O.Hoffm.) H.Rob., Nsaku nsaku, Nsalukiayakala, Salu kia luyakala (Kik.), Salu 44157	Aborting	R	Maceration	Bath	M	1	5
	Backache	R	Chewing	Oral	M	1	
	Constipation	R	Decoction	Oral	M	1	
	Fever	L	Decoction	Steam Bath	M		
	Infertility	L			M	1	
	Parasitic worms	R	Maceration	Oral	M	1	
	Tea	L			N	1	
*Harungana madagascariensis* Lam. ex Poir., Fitila, Leka, Ntunu, (Kik.), Kitunu, Mtunu 43268	Appendix	B	Decoction	Enema	M	1	9
	Construction	WO			D	1	
	Diarrhea	L	Crudité	Oral	M	1	
	Headache	L		Oral	M	1	
	Hepatitis	L	Balm	Enema	M	3	
	Hepatitis	B		Enema	M	2	
	Housebuilding	WO			D	1	
	Menstruation	B	Decoction	Enema	M	1	
	Open cervix	L	Balm	Enema	M	1	
	Skin disease	B	Decoction	Enema	M	1	
	Skin disease	L		Enema	M	1	
	Splenomegaly	B	Decoction	Enema	M	1	
	Stain	LA			H	1	
	Yellow fever	B	Decoction	Enema	M	1	
*Heinsia crinita* (Afzel.) G.Taylor, Nsiamuna*,* Nsangumuni (Kik.) 43181	Anorexia	B	Maceration	Oral	M	1	2
	Constipation	B	Maceration	Oral	M	1	
	Cough	R	Maceration	Oral	M	1	
	Fever	R	Maceration	Oral	M	1	
	Stomachache	B	Maceration	Oral	M	1	
*Helichrysum globosum* Sch.Bip., Mpemba (Kik.) 43892	Stomach pains	R	Decoction	Enema	M	2	2
*Helichrysum mechowianum* Klatt, Kutu kua ngô (Kik.) 42728	Otitis	L	Incinerate	Ear Drop	M	1	2
	Toilet paper	L			O	2	
*Helichrysum* spec.*,* Dimpaludi (Kik.), Dipakula 44116	Earache	R	Percolation	Ear Drop	M	1	3
	Parasitic worms	R	Decoction, Maceration	Enema, Oral	M	2	
*Hibiscus acetosella* Welw. ex Hiern, Usse (Port.) F_48	Anaemia children	L	Decoction, Infusion	Oral	M	2	2
	Heart problems	L	Decoction, Infusion	Oral	M	2	
	Nutrition	L	Decoction, Infusion	Oral	N	3	
*Hilleria latifolia* (Lam.) H.Walter 42612	Nutrition	L			N	1	1
*Hugonia macrocarpa* Welw., Kisilua (Kik.) 43126	Urinary infection	R	Infusion	Oral	M	1	1
*Hymenocardia acida* Tul., Mpete, Mvete (Kik.), lovete, kihete, Vete, kiheta, Iheta 42738	Anaemia	L	Decoction	Bath, Oral	M	2	22
	Bloody diarrhea	R	Maceration, Decoction, Enema	Oral	M	7	
	Cough	L	Crudité, Decoction	Oral	M	2	
	Diarrhea	L	Crudité, Eat	Oral	M	2	
	Diarrhea	B			M	1	
	Fainting	R	Decoction	Inhalation	M	1	
	Hemorrhoids	L	Balm, Decoction	Dermal, Enema	M	3	
	Inflammation legs	R	Decoction	Dermal	M	1	
	Inflammation testicles	S	Decoction	Enema	M	2	
	Lepra	B	Decoction	Dermal	M	2	
	Madness	L, R			M	12	
	Open cervix	B	Crudité	Vaginal	M	2	
	Paralysis	L	Decoction	Dermal	M	1	
	Save pregnancy	B	Crudité	Oral	M	1	
	Scoliosis	L	Decoction	Dermal, Oral, Enema	M	5	
	Sprain	B	Bandage	Dermal	M	1	
	Stimulation	L	Decoction	Oral	M	1	
	Stomach pains	R	Decoction	Oral	M	1	
	Toothache	B	Decoction	Mouth Wash	M	1	
	Weakness	L	Decoction	Oral	M	1	
	Weakness	R	Funge	Oral	M	1	
*Hymenocardia ulmoides* Oliv., Mbanga nvete, Nkalangangula (Kik.), nzangambala, mbangahete 43930	Anaemia	L	Crudité, Decoction	Oral	M	2	10
	Bloody diarrhea	L	Chewing	Oral	M	1	
	Cleaning blood	L	Decoction	Oral	M	1	
	Construction	WO			D	2	
	Cough	L	Crudité, Decoction	Oral, Enema	M	3	
	Diarrhea	L	Chewing, Crudité	Oral	M	2	
	Epilepsy	L	Decoction	Enema, Oral	M	2	
	Scoliosis	L	Balm	Dermal	M	1	
	Sprain	L	Balm	Dermal	M	1	
	Stomach pains	L	Chewing	Oral	M	1	
	Wound	L	Decoction	Dermal	M	1	
*Hypoestes forsskaolii* (Vahl) R.Br.*,* Kimana ngangi (Kik.) 43329	Inflammation	L			M	1	1
	Wounds	L			M	1	
*Hypoxis angustifolia* Lam.*,* Ba dia nseke, Lumpakuludia (Kik.) 44136	Erectile dysfunction	BU, L	Pulverize	Dermal	M	2	2
	Supporting pregnancy	L			M	1	
*Hypselodelphys poggeana* (K.Schum.) Milne-Redh., Mangungu (Kik.), mungungu 41883	Package	L			O	2	2
*Impatiens* spec.*,* Ndaka mboma (Kik.) F_49	Stomachache	W	Crudité	Enema	M	1	1
*Imperata cylindrica* (L.) Raeusch., Sonja (Port.), Nsioni (Kik.) 44098	Stomach pains	R	Decoction	Oral	M	1	2
	Underweight baby	RH	Maceration	Bath, Oral	M	2	
*Indigofera capitata* Kotschy*,* Nkeka za ngô (Kik.), munkando 43364	Breathing problems	L	Decoction	Oral	M	1	2
	Bronchitis	L	Decoction	Oral	M	1	
	Cough	L	Decoction	Oral	M	1	
	Tumor	R	Balm	Dermal	M	1	
*Indigofera erythrogramma* Baker*,* Mbadi mbadi (Kik.) 44015	Stomach pains	R	Chewing	Oral	M	1	1
*Indigofera podocarpa* Baker f. & Martin 44198	Constipation	R	Maceration	Enema	M	1	1
* Inga edulis Mart., Banana macaco (Port.), gazela 44781	Charcoal	WO			D	5	5
	Fodder plant	L			F	1	
	Nutrition	F			N	2	
*Ipomoea prismatosyphon* Welw.*,* Kiniata (Kik.) 43948	Splenomegaly	BU	Decoction	Enema	M	1	1
* Jatropha curcas L.*,* Mpuluka, Sinde dia nkaka (Kik.), umpulukua, Mumpulukua, Puluka, Mupululuka 43845	Dermal infection	RE		Dermal	M	1	9
	Diabetes	R	Decoction	Oral	M	1	
	Diabetes	L	Decoction	Oral	M	1	
	Epilepsy	L	Percolation	Nose Drops	M	1	
	Fencing	W			D	1	
	Inflammation	L	Decoction	Dermal	M	1	
	Malaria	L	Pulverize	Enema	M	1	
	Skin disease	L	Pulverize	Enema	M	1	
	Skin disease	LA		Dermal	M	1	
	Toothache	LA	Decoction	Mouth Wash, Inhalation	M	2	
		L			M	1	
*Kalanchoe crenata* (Andrews) Haw.*,* Ntontozi (Kik.), Luikaika, totozi, mukaikai, kayuki 44194	Against storms (totozimalembozi)	L			R	1	7
	Earache	L	Percolation	Ear Drops	M	2	
	Eye problems	L	Percolation	Eye Drops	M	1	
	Lepra	L, R	Decoction	Enema	M	2	
	Otitis	L	Percolation	Ear Drops	M	1	
	Struck by lightning	L	Crudité	Oral	M	1	
*Lagenaria siceraria* (Molina) Standl. F_50	Bottle	F			O	1	1
*Landolphia camptoloba* (K.Schum.) Pichon*,* Mbungu mbungu (Kik.)*,* nzozu, Mata 44119	Nutrition	F			N	4	4
*Landolphia congolensis*	Nutrition	F			N	1	1
*Landolphia owariensis*	Nutrition	F			N	1	1
*Lannea antiscorbutica* (Hiern) Engl. Nkumbi (Kik.)*,* mukumbi 43179	Diabetes	B	Maceration	Oral	M	1	12
	Eye problems	B	Percolation	Eye Drop	M	2	
	Fracture	B	Balm	Dermal	M	1	
	Hemorrhoids	B	Balm	Dermal	M	1	
	Hemorrhoids	L	Decoction, Maceration	Oral, Dermal	M	2	
	Lazeration	B	Apply On Surface	Dermal	M	1	
	Leg injury	B	Balm	Dermal	M	2	
	Sprain	B	Bandage, Decoction	Dermal	M	2	
	Toothache	B	Decoction	Mouth Wash, Inhalation	M	4	
	Buculosis	FL, L	Decoction		M	2	
	Wounds on chest	FL, L	Decoction		M	2	
*Lannea edulis* (Sond.) Engl., Nkumbi (Kik.), kanda, kakumbi 44158	Bloody diarrhea	R	Decoction, Maceration	Oral	M	2	4
	Colorant	R			O	1	
	Diarrhea	L, R	Decoction		M	2	
	Erectile dysfunction	R	Chewing	Oral	M	1	
	Strong diarrhea	R	Crudité	Oral	M	1	
*Lannea welwitschii* (Hiern) Engl.*,* Nkumbi (Kik.) 43832	Scoliosis	B	Decoction	Dermal	M	1	2
	Toothache	B	Decoction	Inhalation	M	1	
*Lantana angolensis* Moldenke*,* Bulukutu (Kik.) 44740	Tea	L			N	1	1
* Lantana camara L. 43374	Cough	L	Infusion	Oral	M	1	2
	Obstructed airways	L	Infusion	Oral, Steam Bath	M	2	
	Tea	L			N	1	
*Laportea mooreana* (Hiern) Chew, kahidi 44164	Asthma	L	Decoction	Oral	M	1	1
*Lasimorpha senegalensis* Schott, Tiokola, Tiokuela (Kik.) 44019	Fodder plant	L			F	1	2
	Packaging	L			O	1	
*- Lavandula angustifolia* Mill.	Menstrual cramps	F	Incinerate		M	1	1
*Leonotis leonurus* (L.) R.Br., Kumba dia mvuala (Kik.) 42874	Epilepsy	L	Maceration	Enema	M	2	3
	Hepatitis	L	Decoction	Oral	M	1	
	Stomach pains	L	Decoction, Maceration	Oral, Enema	M	2	
*Leptactina benguelensis* (Welw. ex Benth. & Hook.f.) R.D.Good*,* Idídi 42825	Nutrition	F			N	1	1
*Leptoderris congolensis* (De Wild.) Dunn*,* Mfundi (Kik.) 42614	Birthing problems	L	Incinerate	Vaginal	M	1	1
*Leptoderris nobilis* var. *Latifoliolata,* Muika wa mbua (Kik.) 43357	Backache	L	Maceration	Enema	M	1	1
*Lippia multiflora* Moldenke, Bulukutu (Kik.)*,* tandela 44101	Appetizing	L	Decoction	Oral	M	1	3
	Cleaning lymph	L	Decoction	Oral	M	1	
	Tea	L	Decoction	Oral	N	1	
*Luffa cylindrica* (L.) M.Roem.*,* Nsanu (Kik.) 42712	Cosmetics	F			T	1	4
	Sponge	F			O	4	
*Maesopsis eminii* Engl., Ntendani (Kik.)*,* mutendani 43904	Backache			Enema	M	1	2
	Splenomegaly	B	Decoction	Enema	M	1	
* Mangifera indica L.*,* Mangueira (Port.), Manga (Kik.) 42871	Anaemia	L	Decoction	Bath, Oral	M	2	7
	Caries	B	Decoction	Mouth Wash	M	1	
	Corn wine production	B			C	1	
	Diarrhea	B	Decoction, Maceration	Oral	M	3	
	Flavour for lunguila	B	Decoction	Oral	C	1	
	Heart problems	B	Decoction	Bath, Oral	M	2	
	Ingredient for wine (lunguila)	B			C	1	
	Nutrition	F			N	2	
	Open cervix	B	Decoction	Bath	M	1	
	Vertiz	B	Decoction	Bath, Oral	M	2	
*- Manihot esculenta* Crantz, Mandioca (Port.), Nsak (Kik.), kisaka 42760	Activates lactation	BU	Crudité	Oral	M	1	5
	Bee repellent	L			F	1	
	Eye parasites	R	Percolation	Eye Drop	M	1	
	Skin disease	L	Decoction	Dermal	M	1	
	Toothache	BU	Incinerate	Dermal	M	1	
*- Manihot glaziovii* Müll.Arg.*,* Nkueza, N´saki (Kik.), mandioca do kongo F_52	Nutrition	L			N	3	3
	Repair	LA			O	1	
*Manotes expansa* Sol. ex Planch.*,* Menga menga (Kik.), mamengamenga 43953	Hemorrhoids	L	Ss		M	1	4
	Nose bleeding	L	Percolation	Nose Drops	M	1	
	Scoliosis	L	Decoction	Dermal, Oral, Enema	M	3	
	Stomach pains	L	Pulverize + Water	Oral	M	1	
*Maprounea africana* Müll.Arg., Kanzonzo, Mbunza, Nsiele nsiele (Kik.) 42808	Constipation	L	Decoction, Eat	Oral	M	4	15
	Constipation	R	Eat	Oral	M	2	
	Constipation	B			M	1	
	Cough	L	Decoction, Eat	Oral	M	2	
	Diabetes	R	Crudité	Oral	M	1	
	Epilepsy	R	Chewing	Oral	M	1	
	Hernia	R	Decoction	Enema	M	1	
	Leg pain	R	Balm	Dermal	M	1	
	Open cervix	L	Crudité	Vaginal	M	1	
	Poor lactation	L	Chewing	Oral	M	2	
	Potbelly	R	Eat	Oral	M	1	
	Scoliosis	R, L	Decoction	Dermal	M	2	
	Toothache	L	Chewing, Crudité, Decoction	Oral, Inhalation	M	5	
	Toothache	R	Decoction	Mouth Wash	M	2	
*Markhamia tomentosa* (Benth.) K.Schum. ex Engl., Nsasa (Kik.) 43915	Eye problems	R	Percolation	Eye Drops	M	1	2
	Infertility	L		Bath	M	1	
	Misfortune	L	Decoction	Dermal	R	1	
*Melanthera scandens* (Schumach. & Thonn.) Roberty, Nkaiala 43972	Injury	L			M	1	1
*Melinis minutiflora* P.Beauv.*,* Malekambua (Kik.) 43896	Fodder plant	W			F	1	1
	Premature contractions (pregnancy)	W		Oral	M	1	
	Stomach pains pregnancy	W		Oral	M	1	
*- Millettia laurentii* De Wild.*,* Pau preto (Port.)	Construction	WO			D	1	1
*Millettia versicolor* Baker, Pau ferro (Port.), M´bota (Kik.), Mbotembandu 43220	Construction	WO, B			D	2	8
	Coppice for fencing	ST			D	1	
	Malaria	L	Decoction	Oral	M	1	
	Rheumatism	L	Apply On Surface	Dermal	M	1	
	Rip pain	B	Tie Around Body	Dermal	M	1	
	Sprain	L	Decoction, Apply On Surface, Balm	Dermal	M	3	
	Tendon strain	L	Decoction	Dermal	M	1	
* Mirabilis jalapa L., jovan, Belle de nuit 43131	Anaemia	L	Infusion	Oral	M	1	2
	Decoration	W			M	1	
	Nutrition	L	Infusion	Oral	N	1	
*Momordica charantia* L., Dimbunzu, Lumbuzua mbuzua, Mambuzu, (Kik.), Mbusuabusua 42620	Antifertil				M	1	8
	Backache	W	Put into Trousers	Dermal	M	1	
	Baso				M	1	
	Childhood disease	L			M	1	
	Clean babys belly	L		Enema	M	1	
	Clean breast milk	L		Enema, Oral	M	2	
	Constipation	L	Maceration	Oral, Bath	M	3	
	Stomach pains	L	Maceration	Enema, Oral	M	4	
*Momordica foetida* Schumach. F_53	Chills	L	Decoction	Oral	M	1	1
*Mondia whitei* (Hook.f.) Skeels, Kimbiolongua, Londolondo (Kik.) 44674	Bodypain	R	Chewing	Oral	M	1	12
	Cough	L	Balm	Dermal	M	1	
	Dental care	R			T	2	
	Erectile dysfunction	R	Crudité, Chewing, Decoction	Oral	M	13	
	Nutrition	L			N	7	
	Tooth cleaning	R, WO			T	1	
	Vegetable	L			N	1	
*Monodora myristica* (Gaertn.) Dunal*,* Mpeve (Kik.) 44707	Aphrodisiac agent	S	Pulverize	Oral	M	1	15
	Appetizing	F, S	Pulverize	Oral	M	2	
	Backache	S, F	Infusion	Oral	M	3	
	Chest pain	R	Decoction	Oral	M	1	
	Cough	S	Balm	Dermal	M	1	
	Cough	R	Decoction	Oral	M	1	
	Debaso	S	Pulverize, Balm	Dermal	M	1	
	Fever	S	Eat, Infusion	Oral	M	2	
	Infertility	S	Decoction	Oral	M	2	
	Inner diseases	B, S		Oral	M	2	
	Kidney	S	Roast	Dermal	M	1	
	Malaria	S	Pulverize, Balm	Dermal	M	1	
	Parasitic worms	B, S		Oral	M	2	
	Premature contractions (pregnancy)	S		Oral	M	1	
	Scoliosis	S, R	Decoction	Dermal	M	2	
	Splenomegaly	S	Decoction	Enema	M	1	
	Sprain	S			M	1	
	Stomach pains pregnancy	S		Oral	M	1	
	Stomachache	S	Roast	Eat	M	1	
	Weakness	R, S	Maceration	Oral	M	3	
*Morinda lucida* Benth., Nsiki (Kik.), masiki, nxiki 42744	Intestinal bacteria	R, L	Decoction	Oral	M	2	8
	Malaria	L, B	Decoction	Oral	M	3	
	Parasitic worms	B, L	Decoction	Oral	M	3	
	Parasitic worms	R	Decoction	Oral	M	1	
	Splenomegaly	L	Decoction	Enema	M	1	
	Sterility (men and women)	L	Decoction	Oral	M	1	
	Stomach pains	L	Decoction	Oral, Enema	M	5	
	Stomach pains	B, R	Decoction	Oral, Enema	M	4	
	Typhus	L, R	Decoction	Oral	M	2	
*Morinda morindoides* (Baker) Milne-Redh.*,* Disu dia lunguenia, Meso-nkama (Kik.), Kongobololo, Nkongobololo 43356	Cleaning blood	L	Decoction		M	1	11
	Parasitic worms	L	Decoction, Infusion	Oral, Enema	M	7	
	Stomach pains	L	Crudité, Decoction	Enema, Oral	M	10	
	Stomach pains	R	Decoction	Oral	M	1	
	Typhus	R, L	Decoction	Oral	M	2	
	Weakness	L	Decoction	Oral	M	1	
*- Morus nigra* L., Doce (Port.), Amoreira F_54	Fodder plant	L			N	1	2
	Nutrition	F			N	2	
*Mucuna pruriens* (L.) DC., Feijao maluco (Port.), Mankundia (Kik.) F_55	Foamy urine	L	Decoction	Oral	M	1	2
	Halluzinogens	L			C	1	
	Soil improvement	W			O	1	
*- Musa × paradisiaca* L., Banana (Port.), Mambuatisa, Mfuka wa dinkongo (Kik.)	Cough	F	Balm		M	1	9
	Diarrhea	F	Cook	Eat	M	1	
	Hemorrhoids	F	Incinerate	Dermal	M	1	
	Nutrition	F, L			N	2	
	Rheumatism	L	Apply On Surface	Dermal	M	1	
	Sanitary pad	L			O	1	
	Skin disease	FL	Balm	Dermal	M	1	
	Toothache	F	Incinerate	Dermal	M	1	
	Traditional banheira	L			D	1	
	Transport	L			H	1	
*Musanga cecropioides* R.Br. ex Tedlie*,* Nsenga nsenga*,* Nsenga (Kik.) *Musenga, musengasenga,* F_75	Birthing problems	L	Decoction	Enema	M	1	6
	Dehydration	R, SS	Drink	Oral	M	1	
	Diarrhea	FL, L	Incinerate	Dermal	M	2	
	Injury	FL, L	Incinerate	Dermal	M	2	
	Revitalization	ST	Drink	Oral	M	1	
	Toothache	B, L	Decoction	Mouth Wash	M	2	
	Weakness	R, SS	Crudité	Oral	M	2	
*Mussaenda arcuata* Poir.*,* Mabolebole*,* Nsiamuna (Kik.) 42654	Anorexia	R	Chewing	Oral	M	1	7
	Hepatitis	L, R			M	2	
	Menstruation problems	R	Infusion	Enema	M	1	
	Nutrition	L, F			N	3	
	Parsitic worms	R	Maceration	Oral	M	1	
	Stomach pains	R	Decoction, Maceration	Oral	M	2	
	Support birth	L	Infusion	Oral	M	1	
		R	Decoction	Oral	M	1	
^*E*^ *Mussaenda nijensis* R.D.Good, Nzamuna 43224	Nutrition	F			N	1	1
*Mussaenda* spec., Nsamuna (Kik.) F_56	Nutrition	F			N	1	2
	Otitis	R	Percolation	Ear Drops	M	1	
*Myrianthus arboreus* P.Beauv., Mbonzo (Kik.), Ntusu, Mbonzu 43174	Nutrition	F			N	2	3
	Yellow fever	L	Decoction	Oral	M	1	
	Yellow fever	ST		Eye Drops	M	1	
*- Newbouldia laevis* (P.Beauv.) Seem.*,* Kafuki, Kavuki, kuvuiti 43913	Fence	ST			D	1	3
	Hemorrhoids	R		Hip Bath	M	1	
	Hemorrhoids				M	1	
	Thrombosis	B	Decoction	Dermal	M	1	
*- Nicotiana tabacum* L.*,* Tabaco de kimbundu (Port.), Mfomo, Tabaco (Kik.), Kizumba, Lulongu 42883	Cigarettes	L			C	3	7
	Cryptorchidism	R	Roast	Dermal	M	1	
	Eye problems	L	Extract Juice	Eye Drops	M	1	
	Eyesight	L	Percolation	Eye Drops	M	1	
	Hernia	L	Balm	Dermal	M	1	
	Sore throat	L	Maceration	Nose Drops	M	1	
	Stomach pains	L, R	Roast	Dermal	M	2	
*Nymphaea lotus* L.*,* Longa dia maza (Kik.) 44143	Infection legs	L			M	1	1
*Ochna afzelii* subsp. *mechowiana* (O.Hoffm.) N.Robson*,* Nkosi nti, Ngonti (Kik.)*,* muhonga 44187	Anaemia	B	Decoction	Oral	M	3	10
	Anaemia	L	Decoction		M	1	
	Backache	B	Decoction	Oral	M	2	
	Body pain	B	Decoction	Oral	M	1	
	Constipation	R	Decoction	Enema	M	1	
	Eye problems	L	Decoction	Steam Bath	M	1	
	Increases blood	B	Decoction	Oral	M	1	
	Lepra	B	Decoction	Enema, Dermal	M	2	
	Paralysis	L	Decoction	Dermal	M	1	
	Scoliosis	L	Decoction	Dermal	M	3	
	Sculptures	WO			H	1	
	Skin disease	B	Decoction	Oral	M	1	
	Sore throat	B, L	Decoction	Steam Bath	M	3	
	Tool handle	WO			D	1	
	Weakness	B	Decoction	Oral	M	1	
*Ochna pygmaea* Hiern*,* Ndombe, Nsosi (Kik.) 44181	Epilepsy	R	Percolation	Eye Drops	M	1	1
	Parasitic worms	R	Percolation	Eye Drops	M	1	
	Splenomegaly	R	Decoction	Enema	M	1	
*Ocimum gratissimum* L.*,* Dinioka nioka, Lumba lumba, Mazudi zudi, Mansusua nsusua (Kik.) 42649	After birth	L	Decoction	Bath	M	1	7
	Cold	L	Decoction	Oral	M	1	
	Flu	L	Decoction	Inhalation	M	1	
	Flu	W	Decoction	Bath	M	1	
	Malaria	W	Decoction	Oral	M	1	
	Malaria	L	Decoction	Inhalation, Enema	M	2	
	Pain	L	Balm, Infusion	Dermal, Oral	M	2	
	Spice	L			N	1	
	Toothache	L	Chewing	Oral	M	1	
					M	1	
*- Oldfieldia africana* Benth. & Hook.f., Mfilu (Kik.) 44731	Vomit	R	Decoction	Enema	M	1	1
*Oncoba dentata* Oliv.*,* Dikaka dia ndianga (Kik.) 42821	Urinal infection	B	Maceration	Oral, Enema, Anal	M	3	1
*Oncoba welwitschii* Oliv.*,* Mbamba (Kik.) 45033	Cold (sniffles)	R, SS	Crudité	Nose Drops	M	1	13
	Constipation	B, F	Decoction	Enema	M	2	
	Construction	WO			D	1	
	Eye pain	R, SS	Crudité	Eye Drops	M	1	
	Fish hunting	F			F	1	
	Fodder plant	L			F	2	
	Headache	L	Infusion	Inhalation	M	1	
	Headache	R	Maceration, Percolation	Nose Drops	M	4	
	Nutrition	F			N	2	
	Paralysis	L	Decoction	Dermal	M	1	
	Parasitic worms				M	1	
	Scoliosis	L	Decoction, Infusion	Dermal, Oral, Enema	M	5	
	Stain	F			H	1	
	Stomach pains	R	Maceration	Nose Drops	M	1	
* Opuntia ficus-indica (L.) Mill.	Nutrition	F			N	1	1
*- Ouratea welwitschii* (Tiegh.) Exell, Kombasesa (Kik.) 44064	Animal trap	L			F	1	2
	Scoliosis	L	Decoction	Bath	M	1	
* Pachira glabra Pasq.*,* Amendoim (Port.), Nguba (Kik.) F_58	Nutrition	S			N	1	1
*Palisota ambigua* (P.Beauv.) C.B.Clarke*,* kudi 44175	Nutrition	L			N	1	1
*Palisota schweinfurthii* C.B.Clarke*,* Mabunda bunda (Kik.), kibundabunda, Pau kisongo F_70	Abrasion	F, L	Decoction	Dermal	M	2	15
	Family problems	F	Crudité	Oral	R	1	
	Gonorrhoea	BU	Decoction	Eat	M	1	
	Impotence	L	Decoction	Oral	M	1	
	Infertility men	R			M	1	
	Inflammation legs	L	Balm	Dermal	M	1	
	Lepra	L	Decoction	Enema, Dermal	M	2	
	Lepra	F	Pulverize	Dermal	M	1	
	Nutrition	ST			N	1	
	Package of cola Ss for storage	L			O	1	
	Paralysis	L	Incinerate	Dermal	M	1	
	Potency	ST, R	Decoction	Enema	M	2	
	Rheumatism	L	Decoction	Dermal	M	1	
	Ritual	L			R	1	
	Scoliosis	L	Decoction	Bath, Dermal	M	2	
	Sexual potency	R		Enema	M	1	
	Sore throat	F	Swallowing	Oral	M	1	
	Tea	ST			N	1	
		L			M	1	
*Palisota ambigua,* kudi 44783	Nutrition	L			N	1	1
*Parinari capensis* Harv., salaki, salakia, salakizu, ikia 42733	Bloody diarrhea	L	Maceration	Oral	M	1	6
	Breathing problems	L			M	1	
	Cough	L	Decoction	Oral	M	2	
	Nutrition	F			N	5	
	Scoliosis	L	Decoction	Enema	M	1	
*Paropsia brazzaeana* Baill., Mbasa*,* Mbasa nseka (Kik.) 43129	Backache	L			M	1	6
	Diarrhea	L	Decoction	Enema	M	1	
	Infertility women	L	Chewing	Oral	M	1	
	Inflammation leg	R	Decoction	Dermal	M	1	
	Leg ache	L, R	Decoction	Oral	M	2	
	Menstruation (severe)	L	Chewing	Dermal	M	1	
	Pain while playing soccer	L	Apply On Surface	Dermal	M	1	
	Scoliosis	L	Decoction	Dermal, Oral, Enema	M	3	
	Stomach pains	R	Decoction		M	1	
	Xibasu = bad magic	R			M	1	
*Parquetina nigrescens* (Afzel.) Bullock*,* Mputumputu (Kik.)	Diarrhea	L	Crudité	Oral	M	1	1
	Stomach pains	L	Crudité	Oral	M	1	
* Passiflora foetida L.*,* 44257	Nutrition	F			N	2	2
*Paullinia pinnata* L. 43906	Activate lactation	L	Decoction		M	1	1
*Pauridiantha mayumbensis* (R.D.Good) Bremek.*,* Simão (Port.) 44014	Scoliosis	L	Decoction	Bath, Dermal	M	2	1
*Pentadiplandra brazzeana* Baill., Hamba (Kik.) 43341	Flatulence	R	Chewing	Oral	M	1	1
	Nutrition	F			N	1	
*Pentarhopalopilia marquesii* (Engl.) Hiepko*,* Nkombo (Kik.) 44176	Constipation	R	Decoction	Enema	M	1	2
	Stomach pain	R	Decoction	Oral	M	1	
*Perichasma laetificata* Miers*,* Kazilingizimue (Kik.) 41875	Epilepsy	R	Crudité	Oral	M	1	2
	Helps solving problems	R	Chewing, Put into Pocket		R	4	
	Protection during war	R			R	1	
*- Persea americana* Mill.*,* Abacate, Abacateiro, Caroso de abacate (Port.), Mavoka, Mvoka (Kik.)	Headache	B, S	Balm	Dermal	M	2	3
	Hemorrhoids	B	Balm	Dermal	M	1	
	Hemorrhoids	B	Decoction	Enema	M	2	
	Stain	F			H	1	
*Petersianthus macrocarpus* (P.Beauv.) Liben, Nsati (Kik.) 44188	Fodder plant	L			F	1	1
* Phaseolus vulgaris L., Makasikila (Kik.), 42758	Nutrition	S			N	1	1
	Nutrition	L			N	1	
*Phyllanthus polyanthus* Pax, Mantomina (Kik.) 44016	Cough	L	Chewing, Pulverize	Oral	M	2	1
*Phyllanthus* spec.*,* Mfunga mfunga (Kik.) 44251	Skin disease	L	Balm	Dermal	M	1	1
* Physalis angulata L., Bulabula, Mabulabula (Kik.) 44793	Stomach pains	L	Maceration	Enema	M	1	2
	Stomach pains baby	L		Enema	M	1	
*Piper guineense* Schumach. & Thonn.*,* Kumpidi (Kik.), Kapidi 44780	Nutrition	F			N	1	4
	Parasitic worms	F		Oral	M	1	
	Spices	F			N	2	
	Stomach pains	F		Oral	M	1	
	Cough	F	Crudité	Oral	M	1	
*Piper umbellatum* L.*,* Lembe*,* Lembe kia mfinda, Malemba lemba*,* Nkángati (Kik.) 42664	Burn	L	Crudité	Dermal	M	1	5
	Chest pain	L	Eat	Oral	M	1	
	Newborn baby crying	L	Colocate In Bed	Dermal	M	1	
	Nutrition	L	Decoction		N	3	
	Support birth	L	Eat	Oral	M	1	
*Piptadeniastrum africanum* (Hook.f.) Brenan*,* Nsinga nsinga (Kik.) 42798	Splenomegaly		Decoction	Enema	M	1	1
* Plantago major L. 43841	Gastritis	L	Decoction	Oral	M	1	1
	Typhus	L	Decoction	Oral	M	1	
*Plectranthus esculentus* N.E.Br.*,* Batata gitamba 42813	Nutrition	BU			N	1	1
*Pleiotaxis rugosa* O.Hoffm.*,* Matita, Ntalamakatesi*,* Telema katesi (Kik.) 43893	Abdominal infect	R	Chewing	Oral	M	1	8
	Anorexia	L	Crudité	Oral	M	1	
	Diabetes	L	Decoction	Oral	M	1	
	Diarrhea	BU, L	Decoction	Oral	M	2	
	Hunters goos luck	R			R	1	
	Infertility men	R	Decoction	Oral	M	1	
	Inflammation testicles	BUs	Decoction	Enema, Dermal	M	2	
	Lack of appetite	R	Chewing	Oral	M	1	
	Parasitic worms	L, BU	Decoction	Oral	M	2	
	Stomach pains	R	Chewing, Decoction	Oral	M	4	
*Plumbago zeylanica* L., Bau bau (Kik.) 42829	Fever	R, L		Bath	M	2	1
	Leg ache	R		Dermal	M	1	
*Pollia condensata* C.B.Clarke, Caldeia (Port.), Kiesekiese, Mpimpita (Kik.) mampipita, pipita 44227	Fraud	S	Swallowing	Oral	R	1	7
	Helps solving problems	S	Put into Pocket, Swallowings	Oral	R	4	
	Pleasure	S			L	1	
	Ritual	S			R	1	
	Splenomegaly				M	1	
*Protea petiolaris* (Hiern) Baker & C.H.Wright*,* Kikumbi kia ngunga, Mbimbi, Mvanga, Sokila (Kik.) 44208	Charcoal	WO			D	2	5
	Diarrhea	R			M	1	
	Fodder plant	L			F	1	
	Headache	L	Balsam	Dermal	M	1	
	Infertility	R	Decoction	Enema	M	1	
	Menstruation problems	R	Decoction	Enema	M	1	
	Rheumatism	L	Decoction	Dermal	M	1	
	Stomach pains	R	Decoction	Enema	M	2	
*Pseudospondias longifolia* Engl., Nviwa (Kik), nviwua, nviua 44199	Anaemia	L	Decoction	Bath, Oral	M	2	4
	Backache	L	Fermentation	Oral	M	1	
	Diarrhea	B	Maceration	Enema	M	1	
	Hemorrhoids	L	Decoction	Oral	M	1	
	Nutrition	F			N	1	
*- Psidium guajava* L.*,* Goiaba, Goiabeira (Port.),Mfuluta (Kik.) 42660	Asthma	L	Infusion	Oral	M	1	8
	Bloody diarrhea	R	Decoction	Oral	M	1	
	Cough	L	Infusion	Oral	M	2	
	Diarrhea	L	Chewing, Crudité, Decoction	Oral	M	6	
	Nutrition	F			N	5	
*Psorospermum febrifugum* Spach*,* Kilengo lengo, Kisoko soko, Lengula, Mfiofio, Mfitila, Nfiofiofio, Nlengula, Nsoko nsoko, Windu wakiana (Kik.), Fiofio, kifitile, Mhotola 42626	Bird trapping	F			F	2	19
	Bleeding	R	Decoction	Enema	M	1	
	Bleeding penis	L	Chewing	Oral	M	1	
	Bloody diarrhea	L	Chewing, Decotion	Oral, Enema	M	2	
	Bloody diarrhea	B	Decoction	Enema	M	1	
	Cough	L	Chewing	Oral	M	1	
	Decoration	F	Put On Bed		R	1	
	Diarrhea (heavy)	L	Chewing	Oral	M	1	
	Eye problems	F	Swallowing	Oral	M	3	
	Heart problems	L	Roast	Oral	M	1	
	Hemorrhoids	L	Decoction	Enema	M	1	
	Lepra	L	Incinerate	Dermal	M	1	
	Lepra (maladimakay)	B	Decoction	Dermal	M	1	
	Nosebleed	R, L	Percolation	Nose Drops	M	2	
	Skin disease	B	Balm, Decoction	Dermal	M	4	
	Skin disease	F, SS, R	Balm	Dermal	M	5	
	Typhus	F	Swallowing	Oral	M	1	
*Psychotria* spec., Nseke nseke (Kik.) F_59	Injury	B			M	1	1
	Toothache	B			M	1	
Pteridium aquilinum subsp. Africanum, Matekua, Manzemba nzelele, Mitekua, (Kik.), kinzelele 42671	Asthma	L	Decoction	Oral	M	1	8
	Leg ache	L	Decoction	Dermal	M	1	
	Nosebleed	L			M	1	
	Nutrition	L			N	6	
	Skin disease	L	Crudité	Dermal	M	1	
	Vertigo	L	Balm, Percolation	Dermal, Nose Drops	M	2	
*Pterocarpus angolensis* DC.*,* Nkula nkula, Ntete mbula, Nkosu (Kik.) 42735	Bloody diarrhea	R	Decoction	Enema	M	1	6
	Children not walking	F	Balm	Dermal	M	2	
	Medicine	R			M	1	
	Menstruation (severe)	R	Decoction	Enema	M	2	
	Save pregnancy	R	Maceration	Enema	M	1	
	Smoking	F			C	1	
*Pycnanthus angolensis* (Welw.) Warb., Muscada (Port.), Banda nzazi, Ndidila, Nlenda, , Munzanga, Nozungu nkumbi (Kik.) 44478	Construction	WO			D	1	7
	Drums	WO			L	1	
	Fodder plant	L			F	3	
	Infertility women	F, B			M	2	
	Spice	S			N	1	
Raphia spec., Bordão (Port.), Matombe, Nkulu (Kik.) F_60	Asthma	F	Incinerate	Oral	M	1	20
	Bottle cork for maruvu can	L			O	1	
	Bronchitis	F	Balm	Dermal	M	3	
	Construction	L			D	4	
	Diabetes	F	Crudité	Oral	M	1	
	Fiber	L			H	1	
	Fish trap	L			H	2	
	Fodder plant	ST			F	3	
	Measles	L	Incinerate	Dermal	M	1	
	Nutrition	F			N	1	
	Palm wine	SS			C	2	
	Parasitic worms	F	Crudité	Oral	M	2	
	Typhus	F	Roast	Eat	M	1	
*Raphia textilis* Welw., Bordão, Maruvo (Port.) F_61	Fiber	L			H	1	1
	Palm wine	SS			C	1	
*Rauvolfia mannii* Stapf, Zumbu dia nkento (Kik.) 43923	Drug	B			C	1	1
*Rauvolfia vomitoria* Afzel.*,* Mvuala, Nzumbu dia kabonzo, Zumbu, Mundungu (Kik.) 42723	Drug	B			C	1	8
	Infertility men	R	Maceration	Enema	M	1	
	Malaria	R	Decoction	Oral	M	1	
	Stomach pains	B	Chewing	Oral	M	1	
	Stomach pains	R	Crudité	Oral, Enema	M	5	
*Renealmia africana* Benth., Dinsasa dia mpumba, Mansansa (Kik.) 43946	Backache	R, F			M	2	
	Malaria	R, F			M	2	
	Nutrition	F			N	1	
*Ricinodendron heudelotii* subsp. *africanum*(Müll.Arg.) J.Léonard*,* Munguela, Monguela (Kik.) 42845	Drum	WO			L	1	
	Fodder plant	L			F	5	
	Headache	L	Balm	Dermal	M	1	
	Nutrition	F			N	1	
	Stomach pains	B	Decoction, Maceration	Enema	M	2	
* Ricinus communis L., Mpanza, Ngono, Mahanzu, Gimono, Mbono (Kik.) 42668	Constipation	S	Chewing	Oral	M	1	6
	Eyes pain	L	Percolation	Eye Drops	M	1	
	Hemorrhoids	L	Decoction	Bath	M	1	
	High blood pressure	L	Balm, Decoction	Dermal, Bath	M	2	
	Inflammation	L	Decoction	Dermal, Bath	M	3	
	Scoliosis	S	Crudité	Dermal	M	1	
	Stomach pains	S	Chewing	Oral	M	1	
*Rothmannia whitfieldii* (Lindl.) Dandy*,* Lubanzi lua mpakasa (Kik.) F_62	Tattoo	F	Extraction		R	1	1
* Saccharum officinarum L., Cana de açúcar (Port.), Cana de lunguila (Kik.)	Wine	SS	Decoction	Oral	C	3	3
*- Sambucus canadensis* L., Mumvumbi (Kik.) 42580	Food aches	L	Balm	Dermal	M	1	1
*Sarcocephalus latifolius* (Sm.) E.A.Bruce*,* Kilolo kia pumba, Lolo, Lolo kia mabundu, Nlolo, Nzelenge (Kik.) 43154	Anaemia	L	Balm, Decoction	Dermal, Bath, Oral	M	3	16
	Antibiotic	R	Decoction	Oral	M	1	
	Diabetes	F	Crudité	Oral	M	1	
	Diarrhea	R	Maceration	Oral	M	1	
	Erectile dysfunction	R	Decoction	Oral	M	1	
	Infertility	R	Decoction	Enema, Oral	M	3	
	Malnutrition	L	Balm	Dermal	M	1	
	Parasitic worms	R	Chewing	Oral	M	3	
	Parasitic worms	B	Maceration	Oral	M	1	
	Revitalization (many diseases)	B, R	Maceration	Oral	M	2	
	Stomach pains	R	Decoction, Maceration	Oral	M	6	
	Stomach pains	L	Decoction	Oral	M	1	
	Stomach pains	F	Eat	Oral	M	1	
	Stomach pains	R, B	Maceration	Oral	M	2	
	Strengthening	R	Maceration	Oral	M	1	
	Typhus	R	Decoction	Oral	M	1	
	Womens infertility	B	Decoction	Oral, Enema	M	2	
		R			M	1	
*-Sarcophrynium prionogonium* (K.Schum.) K.Schum.*,* Folha de kwanga (Port.), Makaya ma kwanga (Kik.) F_63	Baskets	L			H	1	2
	Package	L			O	1	
*Schinziophyton rautanenii* (Schinz) Radcl.-Sm.*,* Dikelekese (Kik.) 44018	Charcoal	WO			D	1	1
Schizophyllum commune, Cogumelo, Turu turu (Port.), Luvua, Kakeketele, Okulokulo (Kik.) F_64	Nutrition	F			N	4	4
*Sclerocroton cornutus* (Pax) Kruijt & Roebers, Ndingambuela, Nguingui mbuela, Ntekele, Nbatekela, Ndingui mbuele, mutekele (Kik.) 43897	Bird trapping	F			F	2	8
	Breathing problems	L, B	Decoction	Oral	M	2	
	Constipation	L			M	1	
	Cough	R, B, L	Chewing, Decoction	Oral	M	12	
	Flu	B	Chewing	Oral	M	1	
	Toys	F			L	1	
*Sclerosperma mannii* H.Wendl.*,* Mamia, Manga (Kik.)	Construction	L			D	1	2
	Construction	L			D	1	
	Roofing	L			D	1	
*Securidaca longipedunculata* Fresen.*,* Nsunda nti, Nsunda (Kik.), Misunda 42740	Backache	R, B	Balm, Decoction	Dermal	M	5	14
	Body pain	R	Balm, Pulverize	Dermal	M	2	
	Breathing problems	R	Decoction	Inhalation	M	1	
	Bronchitis	R	Balsam	Dermal	M	1	
	Cold	R			M	1	
	Constipation	R	Crudité, Decoction	Oral, Enema	M	2	
	Hemorrhoids (internal)	R	Decoction	Oral	M	1	
	Inflammation	R, L, B	Balm	Dermal	M	4	
	Leg ache	R	Balm	Dermal	M	1	
	Muscle cramp	R	Decoction	Dermal	M	1	
	Pain	R	Balm	Dermal	M	1	
	Scoliosis	R, L, B	Balm, Maceration	Dermal, Enema	M	5	
	Stomachache	R	Decoction, Maceration	Enema	M	3	
	Thrombosis	R	Maceration	Enema	M	1	
*Selaginella myosurus* Alston, Malekazanga, Mazangazanga makita (Kik.) 44262	Ritual bath	W			R	1	2
	Scoliosis	W	Decoction		M	1	
* Senna alata (L.) Roxb., Balakasa (Kik.) 44134	Skin disease	L	Balm	Dermal	M	2	2
* Senna occidentalis (L.) Link, Manioka nioka*,* Mansambi nsambi nkau, Nioka nioka (Kik.) 42630	Coffee	S			C	1	13
	Constipation	R	Maceration	Oral	M	1	
	Diarrhea	R	Maceration	Oral	M	1	
	Dysentery	L	Crudité	Oral	M	1	
	Infertility women	R	Decoction	Oral	M	1	
	Kidney problems	L	Maceration	Enema	M	1	
	Liver problems	R			M	1	
	Loss of appetite	R	Crudité, Decoction	Enema	M	3	
	Respiratory problems	S	Roast	Oral	M	1	
	Stomach pains	R, L	Chewing, Crudité, Decoction, Maceration	Oral, Enema	M	15	
	Stomach pains baby (first 2 weeks)	L		Enema	M	1	
	Vomit	R	Maceration	Oral	M	1	
* Senna septemtrionalis (Viv.) H.S.Irwin & Barneby, Malulu (Kik.), Malulua 43177	Stomach pains	L		Enema	M	1	1
*Sesamum indicum* L., Bulukutu, Wanguila (Kik.) 43895	Cicatrices	S	Oil	Dermal	M	1	2
	Inflammation	S	Oil	Dermal	M	1	
	Nutrition	S			N	1	
	Pain	W	Decoction	Inhalation	M	1	
	Thromboses	S	Oil	Dermal	M	1	
	Weakness	W	Decoction	Inhalation	M	1	
*Sesamum radiatum* Schumach. & Thonn.*,* Wandu wandu*,* Wanguila (Kik.) 42742	Blemished skin	L	Decoction	Bath	M	1	2
	Typhus	L	Decoction	Enema	M	1	
*Sesbania sesban* (L.) Merr., Nkuamba (Kik.)*,* Kuanda 42632	Induced abortion	R	Crudité	Enema	M	1	1
*Setaria megaphylla* (Steud.) T.Durand & Schinz*,* Capim do diabo (Port.)*,* Kangaya, Makangaya (Kik.) 43246	Arm pain	L	Balm	Dermal	M	1	5
	Fodder plant	L			F	2	
	Supports birth	L	Maceration	Oral	M	1	
	Urinary bladder pain	R	Decoction	Oral	M	1	
*Sida urens* L., Lumvumbu (Kik.) F_65	Madness	L	Percolation	Nose Drops	M	1	1
*Smilax anceps* Willd., Mpolo*, N*gila ngila*,* Nzila nzila (Kik.) 43197	Bloody diarrhea	L, R	Crudité, Decoction	Oral	M	4	13
	Cough	L	Decoction	Oral	M	2	
	Epilepsy	L	Percolation	Eye Drops	M	1	
	Erectile dysfunction	R	Chewing	Oral	M	2	
	Hernia	R		Enema	M	1	
	Infection legs	L	Balm	Dermal	M	1	
	Inflammations	L			M	1	
	Neck pain	L			M	1	
	Skin disease	L	Balm	Dermal	M	1	
	Skin disease	F	Eat, Swallows	Oral	M	2	
	Tea	L	Decoction		N	1	
	Vertigo	L	Balm, Percolation	Dermal, Nose Drops	M	2	
		R	Decoction		M	1	
* Solanum aethiopicum L.*,* Mbolongwa (Kik.) 43113	Nutrition	F			N	1	1
* Solanum americanum Mill.	Parasitic worms	F	Chewing	Oral	M	1	1
	Stomach pains	F	Chewing	Oral	M	1	
*Solanum macrocarpon* L.*,* Couve preta (Port.), Lezo (Kik.) 44099	Nutrition	L, F			N	2	2
* Solanum mauritianum Scop.*,* Daniel, Malulua branca 44682	Appendix	L	Decoction	Enema	M	1	3
	Constipation	L	Decoction	Enema	M	1	
	Knee pain	L	Apply On Surface	Dermal	M	1	
	Measles				M	1	
	Stomach pains	L	Decoction	Enema	M	1	
* Spondias mombin L., Gajajeira, Gajaja (Port.), Mungiengie (Kik.) 42879	Diarrhea	B	Decoction		M	1	4
	Eye problems	B	Percolation	Eye Drop	M	1	
	Fence	ST			D	2	
	Nutrition	F			N	1	
	Yellow fever	L	Decoction	Bath	M	1	
*- Stachytarpheta cayennensis* (Rich.) Vahl*,* Agua de joelho (Port.) 42710	Chest pain	W	Decoction	Oral	M	1	5
	Clean lungs	W	Balm	Dermal	M	2	
	Inflammations	W	Balm	Dermal	M	1	
	Knee pain	W	Balm	Dermal	M	1	
	Shoulder pain	L	Apply On Finger	Dermal	M	1	
	Skin disease	W	Decoction	Oral	M	1	
	Typhus fever	L	Decoction	Oral	M	1	
	Weakness	L	Crudité	Oral	M	1	
*Steganotaenia araliacea* Hochst., Mumvumbivumbi, Nkula mvumbi (Kik.), kitomona, Mukala mvumbi 43172	Analgesic	R	Decoction	Oral	M	1	7
	Backache	L	Decoction	Oral	M	3	
	Bad dreams	L			R	2	
	Body pain	L	Maceration	Bath	M	1	
	Cold	R	Dedoction	Oral	M	1	
	Gonorrhoea	R	Maceration	Oral	M	1	
	Insomnia	R	Maceration	Oral	M	1	
	Refreshment	B	Maceration	Oral	M	2	
	Scoliosis	R	Decoction	Dermal	M	1	
	Stomach pains	R	Dedoction	Oral	M	1	
*Sterculia quinqueloba* (Garcke) K.Schum.*,* Mulendi (Kik.) 44732	Backache	L	Roast	Dermal	M	1	1
	Construction	WO			D	2	
	Fiber plant	B			H	1	
	Fodder plant	L			F	1	
	Inflammation	L	Roast	Dermal	M	1	
*Sterculia tragacantha* Lindl.*,* Milenda, Ntutu (Kik.) Nkombolokia, kombolokia 44004	Fire WO	WO			D	1	6
	Fodder plant	L			F	1	
	Handicraft	F			R	1	
	Hemorrhoids	B	Decoction	Oral	M	1	
	Prenatal infection	B	Maceration	Enema	M	1	
	Urinal infection	B			M	1	
*Stomatanthes africanus* (Oliv. & Hiern) R.M.King & H.Rob., Kisalu kia kento, Nkutakani*,* Salu kialukento (Kik.) 44748	Backache	L, W	Decoction	Enema	M	2	4
	Erectile dysfunction	L	Decoction	Oral	M	1	
	Fatigue	L	Decoction	Oral	M	1	
	Good development of baby during pregnancy	R			M	2	
	Heart problems	L	Decoction	Oral	M	1	
	Infertility	L			M	1	
	Supports pregnancy	L	Decoction	Oral	M	1	
*Strophanthus welwitschii* (Baill.) K.Schum., Luvuma, Mvuma (Kik.) 42757	Talisman	F			R	1	2
					M	1	
*Strychnos cocculoides* Baker, Maboque (Port.), Kahole 44124	Baso children	F, R		Enema	M	2	9
	Cryptorchidism	R	Maceration	Oral	M	1	
	Diarrhea	F	Crudité	Eat	M	1	
	Drinking vessel	F			H	1	
	Flute	F			L	1	
	Hernia	R, F	Chewing	Oral	M	2	
	Nutrition	F			N	8	
	Parasitic worms	R	Maceration		M	1	
	Stomach pains	R	Chewing, Decoction, Maceration	Oral, Enema	M	4	
	Stomach pains	F	Chewing	Oral	M	1	
*Strychnos pungens* Soler.*,* Mabumi, Mbumi (Kik.), Kahola muanda, mbitu 43884	Hernia	F	Decoction		M	1	3
	Pain after birth	L	Leaves Put into Clothes, Decoction	Dermal, Enema	M	2	
	Rat trap	F			F	1	
	Skin disease	B	Balm	Dermal	M	1	
	Stomach pains	F	Decoction		M	1	
*Symphonia globulifera* L.f., Ntadia ngombo (Kik.) 43924	Cough	L	Chewing	Oral	M	2	2
	Witchery	LA			R	1	
*Synsepalum cerasiferum* (Welw.) T.D.Penn.*,* Nsuama nkima (Kik.)*,* musuamankima 43283	Backache	B		Enema	M	1	1
*Syzygium guineense* (Willd.) DC., Nkizu, lungama (Kik.) 44138	Bloody diarrhea	R	Decoction	Oral	M	1	12
	Diarrhea	B, L, R	Maceration, Decoction	Oral	M	4	
	Fodder plant	L			F	3	
	Nutrition	F			N	8	
	Parasites in eyes	B	Percolation	Eye Drops	M	1	
	Protection of eyes	L			R	1	
	Stomach pains	B	Decoction	Oral	M	1	
	Toothache	B	Decoction	Inhalation	M	1	
	Typhus	B	Decoction	Oral	M	1	
*- Syzygium jambos* (L.) Alston F_66	Nutrition	F			N	1	1
*Tabernaemontana crassa* Benth., Ngavua za mputu (Kik.) 42823	Construction	WO			D	1	3
	Hiccup	B, L	Maceration	Oral	M	2	
	Snakebite	F, LA	Extraction	Dermal	M	1	
*Tagetes minuta* L., Nkamansongo (Kik.) 42576	Flu	L	Decoction	Steam Bath	M	1	1
*Tapinanthus dependens* (Engl.) Danser, Kikunda 42763	Headache	L, ST	Decoction	Steam Bath	M	2	1
*Tephrosia vogelii* Hook.f.*,* Bualu, Mbaka (Kik.) 42824	Epilepsy	L	Ss	Eye Drops	M	1	5
	Fish toxin	L			F	5	
*Terminalia brachySTma* Welw. ex Hiern*,* mungolo, moeia 44180	Fever	F	Crudité	Oral	M	1	2
	Flu	F	Crudité	Oral	M	1	
	Fodder plant	L			F	2	
	Sore throat	R	Decoction	Inhalation	M	1	
	Stomachache	R	Decoction	Enema	M	1	
*- Terminalia catappa* L. F_67	Decoration village	W			R	1	1
*- Tetracera stuhlmanniana* Gilg*,* Nkudi a nkayi (Kik.) 43833	Nutrition	SS			N	1	1
*- Thonningia sanguinea* Vahl*,* Langa dia mfinda, Mbengela, Timba timba (Kik.), pisa de maluca 43263	Cough	RH	Chewing, Maceration	Oral	M	2	9
	Erectile dysfunction	RH	Chewing, Decotion	Oral	M	9	
	Parasitic worms	RH	Crudité	Oral	M	1	
	Stomachache	RH	Crudité	Eat	M	1	
	Urinal infection	RH	Syrup	Oral	M	1	
*Thunbergia lancifolia* T.Anderson*,* Malavu dia nsongui, Malavu masonguia (Kik.), mpandazeka 44011	Constipation	R	Maceration	Enema	M	1	4
	Hernia	R	Decoction	Enema	M	1	
	Parasitic worms	R	Decoction	Enema	M	1	
	Stomach pains	R	Maceration, Decoction, Chewing	Oral, Enema	M	4	
* Tithonia diversifolia (Hemsl.) A.Gray*,* Malulua, Malulu (Kik.) 44090	Constipation	L		Enema	M	1	1
	Decoration graveyard	FL			R	1	
	Parasitic worms	L		Enema	M	1	
*Trema orientalis* (L.) Blume*,* Ndia nuni (Kik.)*,* Mudianuni, mezendenguenia, yanuni 44216	Fodder plant	L			F	2	3
	Heartache	L, B	Decoction	Oral	M	2	
	Host plant	L			F	1	
*- TriStma leiocalyx* Cogn., Banana de deus (Port.), Kimbunga mbunga, Mbunga mbunga (Kik.). makondo makambolo F_73	Nutrition	F	Crudité	Oral	N	4	4
*Triumfetta cordifolia* A.Rich.*,* Luvunga, Mpunga, Mvungila, Ngongi (Kik.), gigonge, punguila, mpunguele, xipunga, kivungala 42650	Baskets	ST			H	1	8
	Fiber	ST			H	2	
	Fish trap	ST			F	1	
	Jute sack	ST			H	2	
	Rope	ST			H	3	
	Skin problem	R	Balm	Dermal	M	1	
*- Uapaca vanhouttei* De Wild., musambi 44171	Fodder plant	L			F	1	1
*Uraria picta* (Jacq.) DC., Zumbu (Kik.) 42629	Male potency	R			M	1	2
	Stomach pains	R, BU	Crudité	Enema	M	2	
*Urena lobata* L., Kikulokoso, Kolokoso, Lunzunzu, Makolokoso (Kik.), punga 43227	Backache	L	Balm	Dermal	M	1	11
	Constipation	R	Decoction	Oral	M	1	
	Dysentery	L		Enema	M	1	
	Flatulence baby	L		Enema	M	1	
	Healing umbilicus of newborn	L	Apply On Surface	Dermal	M	1	
	Hemorrhoids	R	Crudité		M	1	
	Kidneys	L	Balm	Dermal	M	1	
	Nosebleed	R	Crudité		M	1	
	Pregnancy hard belly		Crudité	Oral	M	1	
	Rope	ST			H	2	
	Stomach pains children	L	Pulverize	Enema	M	1	
	Stomach pains while birth	L	Crudité	Oral	M	1	
	Wounds	L	Balm	Dermal	M	1	
*Vangueria infausta* Burch., mamuemuita 43857	Nutrition	F			N	1	1
*- Vernonella subaphylla* (Baker) H.Rob. & Skvarla, Makútula 42795	Inflammation finger	L		Dermal	M	1	1
	Typhus fever	R			M	1	
*Vernonia amygdalina* Delile*,* Malulu (Kik.) 43285	Dermal infection	BU	Balm	Dermal	M	1	5
	Constipation children	L	Maceration	Enema	M	1	
	Dermal allergy	L	Balm	Dermal, Bath	M	3	
	Malaria	L	Crudité		M	1	
	Parasites	L	Infusion	Oral	M	1	
	Parasitic worms	L	Infusion, Decocton Balm	Oral, Dermal	M	3	
	Stomach pains	L	Decoction		M	1	
*- Vernonia jaegeri* C.D.Adams, Matita (Kik.) 43888	Stomach pains	R	Crudité	Enema, Oral	M	2	1
	Skin infection	BU	Balm	Dermal	M	1	
*Vitex doniana* Sweet, Mfilu (Kik.)*,* mulolo, Filufilu, nzulozulo, Mafilu 43366	Backache	L	Decoction	Oral	M	1	9
	Constipation	R	Decoction	Enema	M	2	
	Cough	L	Decoction	Oral	M	1	
	Diarrhea	R	Maceration	Oral	M	1	
	Epilepsy	R			M	1	
	Fatigue	B, L	Decoction	Oral	M	2	
	Flu	L	Decoction	Inhalation	M	1	
	Headache	L	Decoction	Inhalation	M	1	
	Hemorrhoids (internal)	L	Decoction	Oral	M	1	
	Nutrition	F			N	1	
	Scoliosis	R, L	Decoction	Enema, Bath	M	4	
	Stomach pains	R	Decoction	Enema	M	1	
	Tea	L			N	4	
	Thrombosis	R	Decoction	Dermal	M	1	
*Vitex madiensis* Oliv.	Anaemia	L	Decoction, Infusion	Bath, Oral	M	3	20
	Antibiotic	L	Infusion	Oral	M	1	
	Back pain	L	Decoction, Infusion	Oral, Dermal	M	3	
	Backache	B	Decoction	Oral	M	1	
	Bloody diarrhea	R, L	Decoction	Oral	M	2	
	Body aches	L	Infusion	Dermal	M	1	
	Ceremony	L			R	1	
	Chest pain	R	Percolation	Nose Drops	M	1	
	Cleaning	L	Decoction	Oral	M	1	
	Cough	L	Decoction	Oral	M	1	
	Diabetes	L	Decoction	Oral, Enema	M	21	
	Epilepsy	L	Decoction	Enema	M	1	
	Eye problems	R	Squeeze	Eye Drops	M	1	
	For children during mango season	L	Infusion	Oral	M	1	
	Headache	L	Infusion, Decotion	Dermal	M	2	
	Headache	R	Percolation	Nose Drops	M	1	
	Nutrition	F			N	2	
	Nutrition	R	Roast		N	1	
	Parasitic worms	L	Maceration	Oral	M	1	
	Ritual to overcome illness	W			R	1	
	Scoliosis	L	Decoction	Dermal, Oral, Enema	M	4	
	Sterility (men and women)	L	Decoction	Oral	M	1	
	Stimulation	L	Decoction	Oral	M	1	
	Stomach pains	L	Decoction	Oral, Enema	M	4	
	Tea	L	Infusion	Oral	N	3	
	Weakness	B, L	Decoction	Oral	M	2	
*Xylopia aethiopica* (Dunal) A.Rich., Nkuwa nkuwa, N´sanu (Kik.), nkuakua, Mvamba kuakua F_71	Aphrodisiac agent	F	Pulverize	Oral	M	1	16
	Aromatization	B, F			N	2	
	Asthma	F	Crudité	Oral	M	1	
	Backache	F	Crudité	Oral	M	2	
	Chest pain	R	Decoction	Oral	M	1	
	Childhood disease: Kikongo	F	Pulverize, Balm	Dermal	M	1	
	Construction	WO			D	2	
	Cough	F	Crudité	Oral	M	1	
	Cough	R	Decoction	Oral	M	1	
	Debaso	F	Pulverize, Balm	Dermal	M	1	
	Flavour for meal	F			N	1	
	Infertility men	F	Decoction	Oral	M	1	
	Infertility women	S	Roast	Oral	M	1	
	Inflammation testicles	F	Decoction	Enema	M	2	
	Kidney	F	Roast	Dermal	M	1	
	Malaria	F	Pulverize, Balm	Dermal	M	1	
	Mixture component	F			M	1	
	Parasites in stomach	F	Decoction	Oral	M	1	
	Scoliosis	F, R	Decoction	Dermal, Oral	M	2	
	Skin problems	R, S	Balm	Dermal	M	2	
	Splenomegaly	F	Decoction	Enema	M	1	
	Sprain	F			M	1	
	Stomach pains	S	Pulverize	Oral	M	1	
	Universeal remedy	F			M	1	
	Weakness	F	Maceration	Oral	M	3	
*Xyris* spec., Capim (Port.) F_72	For tattooing	L			R	1	1
*Zanthoxylum gilletii* (De Wild.) P.G.Waterman*,* Nkongo mayeno (Kik.) F_68	Bottle cork	ST			O	2	4
	Injury foot	ST	Balm	Dermal	M	2	
	Toothache	B	Crudité	Dermal	M	1	
* Zea mays L., Milho (Port.), Masangu, Nzemvo za masangu (Kik.), maiz	Aids	F	Infusion	Oral	M	1	5
	Flavour for lunguila	S	Decoction	Oral	C	1	
	Testicle pain	F	Infusion	Oral	M	1	
	Urinary stone	F	Decoction	Oral	M	1	
	Vertigo	S	Balm	Dermal	M	1	
	Vertigo	ST	Incinerate	Dermal	M	2	
*- Zingiber officinale* Roscoe, Gengibre (Port.), Tanga wisi (Kik.) F_69	Aphrodisiac agent	RH	Pulverize	Oral	M	1	2
	Backache	RH	Pulverize	Oral	M	1	
	Mixture component	RH			M	1	
	Weakness	RH	Pulverize	Oral	M	1	
	Chest pain	RH	Pulverize	Oral	M	1	

### Data analysis and ethnobotanical indices

All collected data sets were put into a database using Microsoft Excel. Corresponding to the research issue, the use of pivot-tables allowed the systematic processing of the large and detailed data set (nearly 40,000 data fields) to correlate different features with each other. Tableau Software was used to create selected diagrams. The basic structure of use-reports to list the information follows the principle “informant i mentions the use of species s in the use category u” [51, 52]. Out of the collected data, 10 use categories were defined: “medicinal use (M)”; “nutrition, spices and herbal teas (N)”; “domestic and charcoal (D)”; “Hunting, fishing and animal feed (F)”; “dental care and cosmetics (T)”; “drugs and cigarettes (C)”; “handicrafts (H)”; “ludic, childrens’ toys (L)”; and “rituals (R)”. Uses mentioned less than eight times were summarized in “Others (O)”, including soaps, toilet paper, glue or agricultural purpose like soil improvement inter alia. Since the majority of data refers to medicinal plants, this category was differentiated into 41 secondary categories according to the treated illnesses (Table 5). We used this detailed classification to enable later pharmaceutical studies because in this field the local people who provide information are not capable of classifying different subcategories according to modern medicine since ethnobotanical indigenous knowledge in several cases does not clearly distinguish.

Statistical methods were performed to figure out the influence of age, gender, plant habitat, and distance to Uíge city, use categories and application forms to each other. Chi-square test of independence was used to determine whether a significant relation between two variables exists [53]. Using the Checklist of Plants in Angola [1], the proportion of neophytes was determined.

In order to allow comparing recorded data to other studies, the following quantitative ethnobotanical indices were calculated: Relative Frequency of Citations (RFC), Cultural Importance Index (CI) as well as the Informant Consensus Factor (F_IC_) regarding the secondary categories of illnesses. The Relative Frequency of Citations presents the local significance of each plant species and is calculated for each species as the quotient of the frequency of citations (FC) and the total number of informants (N) [54] (Formula 1). Tardío and Pardo-de-Santayana [51] introduced the CI to ensure data of different studies being compared due to versatility of species use. If the species use would be mentioned in every use category, ten in our study, the CI would be this total number of use categories, i.e. also 10 [51]. In case the species is used in just one use category the CI would be equal to the RFC (Formula 2). Since interviews often were conducted in groups of informants, the number of groups (62) instead of the number of informants (162) was used to calculate the indices.

F_IC_ indicates the homogeneity of the knowledge of the informants [55] (Formula 3). Values differ from 0 (no concordance) to 1 (full accordance). High values therefore illustrate that healers use the same species for the treatment of the same illness.

$$ {\mathrm{RFC}}_{\mathrm{s}}=\frac{{\mathrm{FC}}_{\mathrm{s}}}{N}=\frac{\sum \limits_{i={i}_1}^{i_N}{UR}_i}{N} $$

Formula 1: Calculation of the Relative Frequency of Citations (RFC): s = species, FC = Frequency of Citation by one informant; *N* = total number of informants [54].

$$ {\mathrm{CI}}_s=\sum \limits_{u={u}_1}^{u_{\mathrm{NC}}}\sum \limits_{i={i}_1}^{i_{\mathrm{NI}}}\frac{{\mathrm{UR}}_{ui}}{NI} $$

Formula 2: Calculation of the Cultural Importance Index (CI): s = species, u = use categories; *N* = total number of informants, *i* = informants, NC = the number of use categories, UR_ui_ = the use report of informant I in use [51].

$$ {F}_{ic}=\frac{n_{\mathrm{ur}}-{n}_t}{n_{\mathrm{ur}}-1} $$

Formula 3: Calculation of the Informant Consensus Factor (F_ic_): *n*_ur_ = number of use-reports in each use category; *n*_t_ = number of taxa used [56].

Literature available on medicinal applications of the listed plant species were used for comparison: Neuwinger [57], Iwu [58] and Latham and Konda ku Mbuta [11] of which the latter two reported data in the adjacent Democratic Republic of Congo [11, 57, 58]. In the following the term *citation* is used in the same way as *use-report*.

## Results and discussion

### General findings on vegetation of used plants

The heterogeneity of Uíge’s landscapes and vegetation formations is mirrored by a high variability of data. Nevertheless, several tendencies can be postulated. Our study presents 2390 use-reports (Table 1). Three hundred fifty-eight species representing 96 plant families were identified, 17 of them only to genus level. Of these used plant species, 35% were trees, 26% perennial herbs, 16% shrubs, 12% climbers, 10% annuals and less than 1% parasites. In contrast to a study in southern Angola [6] and one in Namibia [59], woody plants are not used more frequently in our study area compared to herbs since herbaceous plants are found all year around due to the humid forest habitats, and because the much shorter dry season results in a higher availability of plants from savannah areas [6, 24]. Apparently, men (13%) use more climbers than women (8%) certainly due to the fact that climbers are a characteristic element of forest and transition zone where men are going to hunt regularly. However the difference is not significant (chi-square test, *P* = 0.108, *χ*^2^ = 2.578). The use patterns of the other growth forms do not differ between genders in contrast to, e.g. in Eastern Tanzania, where women are more responsible for collecting herbaceous plants while men work with arborescent species [60].

Concurrently, 27% are plants growing in different savannah types, 24% in forests, and 21% in the transition zone connecting these two ecosystems. Furthermore, 20% of the used plants are cultivated, 7% were collected in disturbed areas and 1% are water plants. Comparing habitat and growth form data, some features become apparent. Forty-five percent of the forest species are trees, 21% climbers. This proportion is shifting towards the transition zone where 40% are trees and 31% climbers. These often anthropogenically induced forest edges are characterized by a moist climate with a simultaneous high solar radiation imitating natural gaps caused by treefall. As tropical rainforest disturbance increases, relative abundance of climbers increases, as well [61, 62]. In contrast, from the collected plant species of the studied savannah formations, 42% are trees and 32% perennial herbs [24]. Fifty percent of species collected in disturbed areas are annual herbs, which confirms the fact that annuals are typical for disturbed areas [63]. While just three out of 358 mentioned species are endemic to Angola, 71 species are naturalized that is equivalent to one fifth, 73% of which are still cultivated. In total, 15% of all citations refer to these species. This high number is not surprising. Different studies document the integration of introduced plants into the ethnobotanical repertoires of people [7, 64, 65]. In a study in Brazil, Santos et al. [66] even detected that invasive species overall were considered useful more often than non-invasive species. A closer look reveals that the naturalized species do not fill a gap described in Alencar et al. or Medeiros et al. [67, 68]. They make up a small part in all medicinal categories with an average of 14%, with just one exception in the category “fevers, malaria” where they represent 36%. Out of the 53 citations for this disease category, 15 citations are based only on *Chromolaena odorata* (L.) R.M.King & H.Rob*.* (8) and *Dysphania ambrosioides* (L.) Mosyakin & Clemants (7). Although a wide range of species exist to treat stomachache, the most frequently used species is *Senna occidentalis* (L.) Link, introduced from tropical America, and used for various applications worldwide [69].

Angola’s turbulent history as a Portuguese colony and the resulting cultural influences from other Portuguese colonies such as Brazil led to an interchange of plant use and knowledge as for *Nicotiana tabacum* L., which arrived in Africa in the 1600s or *Arachis hypogaea* L., which was incorporated at the same time into African ethnomedical systems [70]. In particular, certain arable crops from the New World were introduced in Angola, especially from the Solanaceae and Euphorbiaceae. Actual international listings and reports on neophytes and invasive species are still very incomplete for Angola [71, 72]. According to the list of invasive species in Eastern Africa [73], 24 species of our study are detected to have invasive potential. Due to our observations in northern Angola, six plant species display an invasive behaviour: *Chromolaena odorata* (L.) R.M.King & H.Rob*.*, *Inga edulis* Mart., *Lantana camara* L.*, Senna occidentalis* (L.) Link*, Solanum mauritianum* Scop.*,* and *Tithonia diversifolia* (Hemsl.) A.Gray. The species of most invasive power *Chromolaena odorata* forms dense thickets in savannah and forest gaps, disrupting forest successions.

Local people are aware that this plant is not native to their region. Different myths surround its arrival suggesting that *Chromolaena odorata* was introduced rather recently [32, 72]. Nevertheless, in terms of its traditional use in our study, it is in 6th position regarding its RFC-value (Table 2).

**Table 2:** List of the 11 species with the highest Relative Frequency Citation (RFC) including habitat, used plant parts (PP), use categories (UC), number of citations (NC), and Cultural Importance Index (CI). Habitat (Hab.): *C* cultivated, *F* forest, *S* savannah. Plant Parts: *B* bark, *F* fruit, *L* leaf, *R* root, *S* seed, *SS* stem sap, *ST* stem, *W* whole plant, *Wo* wood. Use category: *C* drugs and cigarettes, *D* domestic and charcoal, *F* Hunting, fishing and animal feed, *H* handicrafts, *L* ludic, childrens’ toys, *M* medicinal use, *N* nutrition, spices and herbal teas, *R* rituals, *T* dental care and cosmetics, *O* others; *neophyte

Species	Hab.	PP	UC	NC	RFC	CI
*Annona stenophylla* subsp. *cuneata*	S	R, L, F	M, N	50	0.371	0.435
*Hymenocardia acida*	S	B, L, R, S	M	40	0.355	0.355
*Vitex madiensis*	S	B, L, R, F, W	M, N, R	43	0.323	0.468
*Psorospermum febrifugum*	S	B, L, R, F, SS	F, M, R	29	0.306	0.371
*Raphia matombe*	F	F, L, SS, ST	C, D, F, H, M, N, O	24	0.306	0.371
Chromolaena odorata*	S	L, W	M, O	26	0.274	0.290
*Elaeis guineensis*	C	B, F, FL, L, R, SS, S	C, F, H, M, N, O, R	35	0.274	0.387
*Aframomum alboviolaceum*	S	F, L, R, ST	M, N	34	0.258	0.339
*Sarcocephalus latifolius*	S	B, F, L, R	M	32	0.258	0.242
*Smilax anceps*	S	F, L, R	M, N	20	0.258	0.226
*Xylopia aethiopica*	F	B, F, R, S, Wo	D, M, N	33	0.258	0.306

With regard to the species number, the predominant used plant families are Fabaceae (11.7%), Asteraceae (6.1%) and Rubiaceae (5.6%), followed by Apocynaceae, Malvaceae and Euphorbiaceae (4.2%). The distribution of plant families is difficult to discuss without referring to the occurring vegetation units. Our results therefore confirm the mosaic like heterogeneity of the studied area, influenced by Guineo-Congolian rain forests, Zambesian dry evergreen forests, Miombo woodlands and secondary (wooded) grasslands [24]. This shows a respective preference: species from Fabaceae and Asteraceae have a high percentage of used savannah plants (> 50%) while the percentage of forest plants increases within the other families, especially in Rubiaceae (26%).

**Fig. 2:**
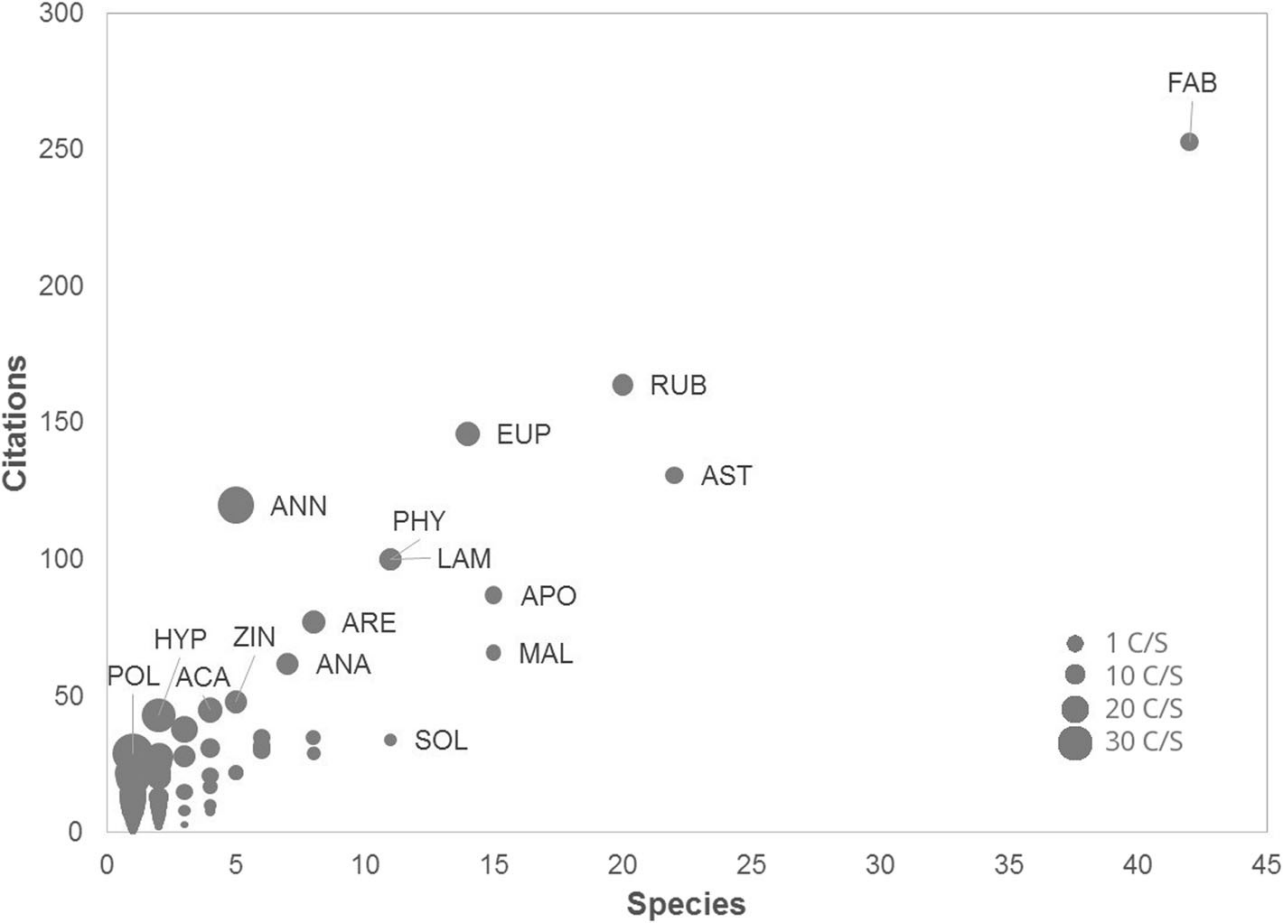
Plant family distribution correlating species number with the number of citations including data about its C/S-Quotient depicted by the size of the circles. Abbreviations of families: ANN Annonaceae, ACA Acanthaceae, ANA Anacardiaceae, APO Apocynaceae, ARE Arecaceae, AST Asteraceae, EUP Euphorbiaceae, HYP Hypericaceae, LAM Lamiaceae, MAL Malvaceae, PHY Phyllanthaceae, POL Polygalaceae, RUB Rubiaceae, SOL Solanaceae, ZIN Zingiberaceae

The quotient of citations and species number within one plant family (C/S) emphasizes the importance of citations within one plant family, including the fact that big families like Fabaceae or Asteraceae inherently show high citation numbers. As illustrated in Fig. 2, some plant families were mentioned with just a few species but high citation rate (high C/S). For example in Annonaceae, 120 citations for 5 species lead to a C/S of 24. While the families Annonaceae and Asteraceae exhibit an equally high number of citations, the number of species is considerably higher in Asteraceae. The proportion for one species therefore is much higher in Annonaceae than in Asteraceae. By contrast, in Solanaceae, 34 citations for 11 species lead to a C/S of 3.1.

### Ethnobotanical results

Willingness of visited people to collaborate was very high. One hundred sixty-two informants were interviewed in 62 groups. Two thirds were older than 40 years. Some healers specialized on one or two diseases only while others demonstrated their broad knowledge to heal a large variety of diseases (Additional file 1).

Seventy-six percent of the citations collected in our study refer to medicinal uses, 10% to nutritional use and 4% to its use as fodder plant. The remaining 10% are divided into the other 7 use categories. Although the unequal split of citations within the 10 use categories suggests a low use of plants in some of them, plenty of species are used for several purposes and daily needs. Thus, 41 species are used for domestic applications, 33 species for rituals, 29 species as drugs or cigarettes, 21 species for handicrafts and 9 for ludic ambits. Compared to other studies (e.g. Vodouhê, [74]), the percentage of medicinal uses is very high, although Göhre et al. [7] detected quite similar use category distributions. One reason might be that our study design required at least one person with knowledge of traditional medicine to accompany the interview. On the other side, this split is an indication of the crucial role of plants in rural health care.

In general, the predominantly used part is the leaf (37%, 890 citations, 220 species), followed by the different stem tissues wood, bark, bast fibres, and resins (17%, 407 citations, 110 species), underground organs like roots, tubers and rhizomes (15%, 367 citations, 140 species) as well as fruits and seeds with 15% (354 citations, 114 species). In some cases, the whole plant (56 citations, 27 species) or flowers (16 citations, 12 species) were used. Regarding only the medicinal use category, the proportion of the citations describing the use of leaves remains almost unchanged with 39% (689 citations, 178 species) while the proportion of the use of underground organs increases by more than the double to 32% (582 citations, 137 species). This detected plant part percentage is consistent with the one observed by Urso et al. [6], Giday et al. [75], or Cheikhyoussef and Embashu, [76]. As already mentioned and discussed in Urso et al. [6], the intensive use of underground organs in medical applications may be due to the fact that underground organs need effective defence strategies based on a high content of secondary metabolites [6, 77]. By contrast, the studies of Upadhyay and Kumar [78] and Panghal et al. [79] confirm leaves as the most frequently used plant part in remedies [78, 79]. In this context, strain and increasing mortality in species of which primarily bark and roots or bulbs are collected for remedies, are discussed [80–83]. No awareness of interviewed people in Uíge province for this emerging problem was detected during our study.

As expected, in the category “nutrition”, the main plant parts used are fruits (57%), leaves (31%), and seeds (5%) which is in concordance with literature [6, 76]. Fruits are consumed fresh, except the fruits of *Adansonia digitata* L., *Piper guineense* Schumach. & Thonn. and *Xylopia aethiopica* (Dunal) A.Rich. which can also be dried. Tubers, although an important source of starch, were seldom mentioned. This may be because during field trips these tubers are not abundant but normally cultivated. For fodder purposes, mainly, leaves are used (75%); fruits and stem tissues only play a subordinate role.

Stems and timber, respectively, are the main plant parts used domestically (67%). Except the significantly more frequent utilization of fruits and seeds by women (chi-square test, *P* = 7 × 10^−5^, *χ*^2^ = 15.8), no other gender-specific difference was detected. This result could be caused by the daily behaviour and responsibilities women have; those inter alia walk to and work in the field, carry and take care of the children while collecting edible fruits along the wayside. For the Ibo women in Nigeria for instance, their ownership of fruit trees was described [84].

### Ethnobotanical indices of used plants

RFC and CI of all mentioned species were calculated to evaluate the importance of the species use. Here, 67% of the species have a RFC below 0.05, 14% between 0.05 and 0.1, and 20% more than 0.1. The values range from 0.37 to 0.02. The species with the highest RFC also show a high variety of its used plant parts and use categories (Table 2). The calculated CI covers values from 0.47 to 0.02 with an average value of 0.07 while Göhre et al. [7] calculated an average value of 0.09 in savannah regions near Uíge city.

Eight of the 11 species listed in Table 2 are typical savannah species demonstrating the importance of this vegetation in traditional plant usage [7]. The most important species is *Annona stenophylla* subsp. *cuneata* (Oliv.) N. Robson, a subshrub, which, due to its woody rhizome, is able to regrow after periodical fires. Fruits are edible, frequent and therefore known by everyone. Its medicinal use is broad but with focus on gastrointestinal disorders. This application was mentioned for the related *A. stenophylla* and *A. stenophylla* subsp. *nana* [57]. *Hymenocardia acida* Tul. as well as *Psorospermum febrifugum* Spach are frequent small savannah trees often used for treating bloody diarrhoea, bleeding or anaemia due to its red root bark producing a reddish coloured decoction and therefore related to blood, according to the tradition of local people. We noticed a comparable relationship between the bark of *Erythrina abyssinica* DC., which produces a yellow decoction and is used to treat yellow fever, and the use of pulverized thorns of the trunk of *Zanthoxylum gilletii* (De Wild.) P.G.Waterman to treat injuries to the feet. Hence, for some plants, appearance is related to functionality, comparable to the doctrine of signatures developed by Paracelsus in the sixteenth century [85, 86]. The shrub *Vitex madiensis* Oliv. produces edible fruits and has a wide variety of healing properties. Another frequent tree is *Sarcocephalus latifolius* (Sm.) E.A.Bruce whose roots are often sold at local markets as a tonic. *Aframomum alboviolaceum* (Ridl.) K.Schum. is a common perennial that produces edible fruits sold at local markets during the late rainy season. *Smilax anceps* Willd. is the only climbing plant in this list, widespread in African savannahs and therefore used diversely [57].

Secondly, three species (*Elaeis guineensis* Jacq*.*, *Raphia matombe* De Wild., *Xylopia aethiopica* (Dunal) A.Rich) are an important part of remedy mixtures and thus quite well known in the literature [57]. Several liposoluble substances can be dissolved in the oil of *Elaeis guineensis* fruits, which is therefore used for skin diseases [57, 58]. At the same time, palm fruits present a food for better nutrition and health due to its components such as palmitic-oleic rich semi solid fat as well as vitamin E, carotenoids and phytosterols [87]. *Xylopia aethiopica* is most commonly used as an addition to remedy mixtures of pulverized seeds due to its diverse constituents [58, 88]. In contrast, the other palm species *Raphia matombe* is one of the most important species in Bakongo culture inter alia because of its common traditional utilization to produce palm wine [89]. In addition, alcohol also serves as solvent for active ingredients [90–92]. Some plants traditionally are macerated in alcoholic beverages and used in medical applications, mainly as aphrodisiac or against pain [57]. However, parts of *Raphia* also serve as base for other applications such as the leaf rachis for domestic use, edible fruits or fibres for handicrafts [32].

Interestingly, the invasive species *Chromolaena odorata*, native to Central-America, is part of the list but also used worldwide for the same purpose or other applications [90, 91, 93–95]. Its biochemical and antimicrobial activities as well as anticancer properties are already well studied [96, 97].

### Ethnobotanical indices of medical plants

If we consider only medical plants, the selected ethnobotanical indices attain values similar to those for the useful plants in general. Out of the 1813 use reports, 68% of the listed plant species exhibit a RFC below 0.05 (corresponding to 3 citations maximal, 13% between 0.05 and 0.1 (4 to 6 citations), and 19% more than 0.1 (from 7 citations up). The values range from 0.34 to 0.02. The species with the highest RFC in this use category are shown in Table 3. Eight of them are already mentioned in Table 3. The calculated CI values range from 0.44 to 0.02 with an average value of 0.08.

**Table 3:** List of 11 medical plant species with the highest Relative Frequency Citation (RFC) including habitat, used plant parts (PP), and Cultural Importance Index (CI). Habitat: *C* cultivated, *F* forest, *S* savannah. Plant parts: *B* bark, *F* fruit, *L* leaf, *R* root, *S* seed, *SS* stem sap, *ST* stem, *W* whole plant; *neophyte

Species	Habitat	PP	RFC	CI
*Hymenocardia acida*	S	B, L, R, S	0.3387	0.4355
*Vitex madiensis*	S	B, L, R	0.3226	0.4032
*Psorospermum febrifugum*	S	B, F, L, R, SS	0.3065	0.3226
*Annona stenophylla* subsp. *cuneata*	S	L, R	0.2903	0.4032
Chromolaena odorata*	S	L	0.2581	0.3226
*Sarcocephalus latifolius*	S	B, F, L, R	0.2581	0.4032
*Aframomum alboviolaceum*	S	L, R, ST	0.2419	0.3226
Dysphania abrosioides*	D	L, R, W	0.2419	0.3226
*Maprounea africana*	S	B, L, R	0.2419	0.3226
*Monodora myristica*	F	B, F, R, S	0.2419	0.371
*Xylopia aethiopica*	F	F, R, S	0.2419	0.3387

Ten percent out of 1813 citations for medicinal uses refer to stomach ache (183 citations), 8% to respiratory diseases (140 citations), 7% to pain and rheumatism (124 citations), 6% to diarrhoea (115 citations) and 6% to headache and weakness (101 citations). According to Heinrich et al. [55], the informant’s consensus can help to select plant species for further pharmaceutical analyses. The calculated Informant Consensus Factor (F_IC_) of the 41 secondary use categories ranged between 0 and 0.78. The disease “measles” has the highest F_IC_ (0.78), followed by the disease groups “diarrhoea” (0.61), “skeletal deformation” (0.6), “anaemia” (0.58) and “stomach ache” (0.58). For 14 out of the 41 defined disease categories, F_IC_ was below 0.2. Table 4 shows the plant species, which were cited at least five times for one disease, sorted by the Informant Consensus Factor (F_IC_) of each disease. Statistical analysis with Chi-square test of independence did not detect any significance in gender-specific treatment of the 41 disease categories, except in the treatment of scoliosis (chi-square test, *P* = 1 × 10^−9^, *χ*^2^ = 37.1).

**Table 4:** Diseases with at least one species mentioned with 5 citations listed in order of its Informant Consensus Factor (F_IC_). In square brackets the number of citations of disease category (UR) and the F_IC_; Known to literature: + known, – not known, *indirectly related; Literature used: Neuwinger, Iwu, Latham and Konda ku Mbuta [11, 57, 58]

Disease	*Species*	UR	L
measles [UR 10; FIC 0.78]	*Gardenia ternifolia* subsp. *jovis-tonantis*	8	–
(bloody) diarrhoea, dysentery [UR 115; F_IC_ 0.61]	*Bridelia ferruginea*	11	+
	*Hymenocardia acida*	10	+
	*Psidium guajava*	8	+
	*Combretum racemosum*	6	+
	*Diplorhynchus condylocarpon*	6	+
	*Elaeis guineensis*	5	+
	*Lannea edulis*	5	+
	*Syzygium guineense*	5	+
skeletal deformation, scoliosis [UR 82; F_IC_ 0.6]	*Aframomum alboviolaceum*	8	–
	*Dialium englerianum*	7	–
	*Hymenocardia acida*	5	–
	*Oncoba welwitschii*	5	–
	*Securidaca longipedunculata*	5	–
anaemia [UR 39; F_IC_ 0.58]	*Annona stenophylla* subsp. *cuneata*	6	–
	*Ochna afzelii* subsp. *mechowiana*	5	+
stomachache [UR 183; F_IC_ 0.58]	*Senna occidentalis*	16	+
	*Morinda morindoides*	11	+
	*Morinda lucida*	10	+
	*Sarcocephalus latifolius*	10	+
	*Annona stenophylla* subsp. *cuneata*	6	+
	*Rauvolfia vomitoria*	6	+
	*Diplorhynchus condylocarpon*	5	+
erectile dysfunction, impotence [UR 59; F_IC_ 0.57]	*Mondia whitei*	13	+
	*Thonningia sanguinea*	9	*
injury, sprain [UR 40; F_IC_ 0.56]	*Chromolaena odorata*	7	+
hepatitis [UR 16; F_IC_ 0.53]	*Erythrina abyssinica*	5	+
skin infection, problems, leprosy, cicatrices [UR 79; F_IC_ 0.5]	*Psorospermum febrifugum*	11	+
	*Chaetocarpus africanus*	5	+
parasitic worms (intestine) [UR 59; F_IC_ 0.48]	*Morinda morindoides*	8	+
	*Morinda lucida*	6	+
fever, malaria [UR 53; F_IC_ 0.48]	*Chromolaena odorata*	8	*
	*Dysphania ambrosioides*	7	+
toothache, caries [UR 44; F_IC_ 0.47]	*Maprounea africana*	7	+
heart problems, blood pressure [UR 18; F_IC_ 0.47]	*Brillantaisia owariensis*	5	–
respiratory diseases [UR 140; F_IC_ 0.47]	*Dysphania ambrosioides*	6	+
rheumatism, gout, pain [UR 124; F_IC_ 0.47]	*Croton mubango*	11	+
	*Securidaca longipedunculata*	9	+
	*Vitex madiensis*	6	+
headache, vertigo, weakness, fatigue [UR 101; F_IC_ 0.45]	*Oncoba welwitschii*	5	+
	*Vitex madiensis*	5	+
eye parasites, eye problems [UR 36; F_IC_ 0.4]	*Albizia adianthifolia*	5	+
epilepsy, convulsion [UR 42; F_IC_ 0.39]	*Costus afer*	5	+
constipation, flatulence [UR 48; F_IC_ 0.34]	*Maprounea africana*	7	+

The importance of traditional medicinal plants is demonstrated by the high number of medical use-reports (76%). This value coincides with those of former studies in this area [7, 9]. The relatively low F_IC_ values could be explained by the heterogeneity of vegetation forms in the studied area. In case of non-availability of one plant species, another will be chosen to treat the same disease. Cheikhyoussef et al. [59] reported much higher F_IC_ due to the considerably lower number of citations, described species and disease categories [59]. The F_IC_ encouraged us to choose reliable data of plants which could be analysed either in medical or phytochemical studies. The majority of the medical applications mentioned at least five times (Table 4) is already known and documented [11, 57, 58], but a few citations are new to science (18%), e.g. *Gardenia ternifolia* subsp. *jovis-tonantis* (Welw.) Verdc. seems to be promising for treatment of measles; except in Göhre et al. [7], the use of *Brillantaisia owariensis* P.Beauv. for cardiovascular diseases was still not documented and *Annona stenophylla* subsp. *cuneata* was neither ethnobotanically nor phytochemically investigated although several studies document the use of related species [7]. With decreasing number of citations the quantity of still unknown uses increases. The disease skeletal deformation/scoliosis is rarely mentioned in ethnobotanical literature as its management is dominated by physiotherapies and bracing and not by herbal preparations. Hulse mentioned deer antlers to cure skeleton deformities according to Chinese medicine and called it of dubious credibility [98]. A study from Namibia mentioned *Ximenia americana* L. as a cure for scoliosis [59]. The standard reference Neuwinger [57] neither mentions skeletal deformation nor scoliosis as traditionally treated diseases [57]. Nevertheless, we documented this traditional healing concept as part of Bakongo health treatment culture.

Administration methods vary from community to community, from healer to healer and from disease to disease. Using a decoction to prepare a remedy is the most frequently found method of preparation (45%), followed by the manufacture of an ointment (13%), maceration (12%) and the application as raw material, while nearly half of all preparations are administered orally (45%), followed by dermal application (20%) in only 16% is an enema used. This is in contrast to commonly used methods used in West African traditional health systems [99]. According to these analyses of administrations, the four most important combinations of preparation and application of medicinal plants are (1) decoction taken orally (21%); (2) raw material crushed, taken orally, chewed or swallowed (14%); (3) maceration of plant parts taken orally (11%); and (4) the preparation of ointment applied to the skin (11%). These findings are in line with those of several studies [6, 7, 100].

### Nutritional plants

Thirty percent of mentioned plant species do have a certain nutritional value for local people. Out of the 107 species used for nutrition, 10 were cited more than five times. Besides the species already listed above (*Aframomum alboviolaceum* (F), *Annona stenophylla* subsp. *cuneata* (F), *Vitex madiensis* (F)), these are as follows: *Anisophyllea quangensis* Engl. ex Henriq. (F), *Dialium englerianum* Henriq. (F), *Mondia whitei* (Hook.f.) Skeels (L), *Parinari capensis* Harv. (F), Pteridium aquilinum subsp. africanum (L.) Kuhn (L)*, Strychnos cocculoides* Baker (F) and *Syzygium guineense* (Willd.) DC. (F).

The use of these species is comparable to Biloso and Lejoly [101], who found very similar results in the province Kinshasa, Democratic Republic of Congo. Termote and Van Damme [102] as well as Latham and Konda ku Mbuta [11] also point out the economic importance of these species. On the other hand, 12% of the citations (13 species) are plants which up to now are not known to literature [7, 9, 11, 103–105]. Especially one species should be highlighted: *Dracaena camerooniana*, whose leaves are locally known as *nsalabayakala*, is also sold at local markets and therefore of economic value. By contrast, fruits like those of *Cnestis ferruginea* or *Renealmia africana* might be edible but not of good taste, so that just a few people do consume these wild fruits, found in the forests. Furthermore, for the consumed aerial parts of *Hilleria latifolia* toxicity studies showed histopathological changes at high doses [106]. As several species are just cited once, further studies on reliability of data as well as on distribution of species, and their nutritive values and toxicities are recommended.

### Influence of gender, age and distance

#### Gender

It is postulated that women and men have separate and unique relationships with biodiversity [37]. Different studies detected either a gender-specific plant use [59, 107] or gender-independent knowledge [108]. In our study, two thirds of informants were male, one third female. In average, female informants concentrate on using plants from savannahs (49%) and villages (38%) while male interviewees focus on the use of forest (40%) and savannah (44%) species.

Although women represent just a fifth of all citations (22%), their contribution to medicinal plants was proportionally even higher (83%) than those of men (74%) (chi-square test, *P* = 9 × 10^−6^, χ^2^ = 19.7). Deleting use categories “medicinal plants” and “nutritional plants”, the remaining use categories can be broken down in detail. It appears that all use categories are nearly homogenously distributed regarding their number of citations between genders and do not differ significantly from each other (chi-square test, *P* > 0.05).

Fifty percent of all plants mentioned in the study were listed just by men, 12% just by women. When looking at more details of the use category “medicinal plants”, a similar pattern can be seen: 48% of the plants were brought up by men only and 14% just by women. The ten most important species mentioned for medical application by women and men, with a percentage of more than 50%, respectively, and the highest numbers of use-reports are shown in Table 5. There is thus a strong suspicion that these species might have a medical application for illnesses specific to women as in Cheikhyoussef et al. [59] or mentioned by Kamatenesi-Mugisha [109]. By contrast, our analyses do not confirm this assumption. Medical plant applications especially for women’s illnesses (menstruation problems, birth, pregnancy, open cervix, lactation, and abortive use) are not significantly more frequently quoted by women than others (chi-square test, *P* > 0.05). On the other hand, mens’ specific illnesses (erectile dysfunction, impotence) and the associated plants are not mentioned just by men, but by women too. On the contrary, percentages are almost evenly distributed.

**Table 5:** List of 10 species representing ≥50% citations of women and men, respectively (%) and highest number of use-reports (UR), with their habitat (H), Habitat: *S* = savanna, *F* = forest, *V* = village; *neophyte

Species mentioned mainly by women	UR	%	H	Species mentioned mainly by men	UR	%	H
*Aframomum alboviolaceum*	15	54	S	*Annona stenophylla* subsp. *cuneata*	32	91	S
*Dialium englerianum*	8	50	S	*Hymenocardia acida*	28	70	S
Jatropha curcas*	7	64	V	*Securidaca longipedunculata*	26	90	S
*Combretum psidioides*	6	86	S	*Sarcocephalus latifolius*	25	78	S
*Ekebergia benguelensis*	6	67	S	*Monodora myristica*	24	80	F
Stachytarpheta cayennensis*	6	67	V	*Vitex madiensis*	23	66	S
*Gaertnera paniculata*	5	100	F	Chromolaena odorata*	22	88	S
*Cola acuminata*	5	63	F	*Psorospermum febrifugum*	22	85	S
Zingiber officinale*	4	100	V	*Bridelia ferruginea*	21	84	S
*Strychnos pungens*	4	80	S	*Morinda lucida*	21	91	S, F

In Bakongo culture, both sexes play a plurality of roles. Nevertheless, a majority of men hunts while women maintain the household, take care of the children and work in the field. However, individual differences from person to person blur these culturally not strictly fixed boundaries, so that men also help on the fields. The results of our study on the influence of gender on plant usage in all areas of daily life did not show prominent differences of genders in traditional plant usage of Bakongo tribes. Handicraft and house constructing activities are performed by both sexes, depending on the transfer of knowledge within the families rather than on gender. Not even in the context of gender-specific illnesses, significant differences could be detected. That all adds up to the conclusion that treatment of illnesses is open and pragmatic and not biased by gender. This notion also contradicts the self-perception of male healers who “use plants of whose women do not know their effects”. But further studies should be undertaken to support this observation, also because the percentage of women was low.

### Distance to Uíge city

As the study was conducted in the whole province covering an area of 59,000 km^2^, different vegetation zones are included which merge together seamlessly forming a complex mosaic. For this reason, it is difficult to detect a clear influence of the distance in regard to species composition in traditional healer’s concepts. What could be detected significantly with respect to the distance to Uíge city are differences in two use categories. The larger the distance, the higher the number of use citations of medical plants ranging from 72% (zone A) to 80% (zone B) (chi-square test, *P* = 9 × 10^−6^, χ^2^ = 19.6) while the use of nutritional plants decrease from 12% (zone A) to 8% (zone B) (chi-square test, *P* = 0.002, χ^2^ = 9.6). Neither plant part utilization nor medical plant explanation or age of informants was significantly different. With increasing distance from the city Uíge and its manifold offers of modern society such as health centres or supermarkets, no significant difference of plant usage could be detected (chi-square test, *P* > 0.05). Similar results were achieved by Ávila et al. [64] who, depending on different urbanization levels, documented the maintenance of a similar ethnobotanical repertoire in Brazilian Quilombola groups. In contrast, Pirker et al. [110] stated an influence of rural–urban urbanization and globalization processes on traditional knowledge. This should be more fully investigated, especially in accordance with the shifts from traditional healing to modern health care in Angola.

### Age

Nearly one third of informants were younger than 40 years whereas only a quarter of all citations were mentioned by this group. The older people therefore show a significantly greater knowledge (chi-square test, *P* = 0.000955, *χ*^2^ = 10.913). Especially concerning the use category “medicine”, significantly more uses were mentioned by the older people (chi-square test, *P* = 0.00097, *χ*^2^ = 10.877). Voeks [107] described a similar situation in northeast Brazil and justified his results to show that the greater knowledge of plant medicinal properties was linked to the greater age of the participant. The reason that the number of young healers is comparatively low is explained by the slow process of transferring knowledge from one generation to another [59]. Further studies should compare firstly younger people and secondly people from urban and rural areas, regardless of their knowledge.

## Conclusion

Despite (or because of) the long-lasting military conflict in Angola, traditional knowledge of plant usage is still an important part of cultural heritage. Plants therefore are essential elements in all areas of livelihood, especially in the medical sector. This situation is compounded by the still very poor health care system in the country, especially in rural areas.

The study reveals the following key messages:

1. A considerable heterogeneity in plant usage of the studied area could be detected, influenced by the high complexity of flora composed of both, Guineo-Congolian and Zambesian elements and the diverse topography.
2. Although the area is large, no significant influence of the distance in regard to species composition in traditional healer’s concepts of the respective village was found.
3. Although several plants were just mentioned by women or men, respectively, no significant restriction to gender-specific illnesses in medical plant use could be found.
4. Merely concerning the age of informants a slight shift could be detected, because one third of informants were younger than 40 years whereas only one fourth of all citations were mentioned by this group. Regarding the analysis within use categories, this tendency could not be substantiated significantly.
5. At least three species are worth evaluating for their pharmacological potential due to their high F_IC_ value regarding the following diseases: *Gardenia ternifolia* subsp. *jovis-tonantis* seems to be promising for treatment of measles; *Brillantaisia owariensis* has still not been analysed for treating cardiovascular diseases; *Annona stenophylla* subsp. *cuneata* was mentioned for treating anaemia.

People in Angola still depend very much on the natural environment, and the knowledge of how to use plants in their daily life is fundamental—even people living in the large cities or urban areas do have family in the rural regions or at least have lived part of their life there. But by virtue of the already existing and for the future expected urbanization and the resultant loss of direct dependence upon nature, traditional knowledge is expected to be lost in future [111], especially if taking into account that Angola has a high amount of unused land, suitable for crops which will be converted in near future, resulting in a negative impact on biodiversity [112]. The study therefore at the same time provides an important contribution of traditional knowledge documentation, which so far is very rare for the area investigated here. Collected data are a worthwhile base for the establishment of a Botanical Garden integrated in the Universidade Kimpa Vita in Uíge with focus on useful plants. Furthermore, ethnopharmacological studies of several selected plant species might usefully be undertaken.

## Additional file

Additional file 1: Short movie: Two of the authors during field studies in Uíge. A traditional healer demonstrates the preparation and application of an herbal funnel. (MOV 142656 kb)

## Abbreviations

CI: Cultural Importance Index
COI: Herbarium Coimbra
F_IC_: Informant Consensus Factor
LISC: Herbarium Lisbon
RFC: Relative Frequency of Citations
